# Structure and Surface Relaxation of CeO_2_ Nanoparticles Unveiled by Combining Real and Reciprocal Space Total Scattering Analysis

**DOI:** 10.3390/nano12193385

**Published:** 2022-09-27

**Authors:** Marco Scavini, Federica Bertolotti, Jonadri Mlloja, Filippo Umbri, Anna Bosc, Serena Cappelli, Stefano Checchia, Cesare Oliva, Patrizia Fumagalli, Davide Ceresoli, Mariangela Longhi, Antonietta Guagliardi, Mauro Coduri

**Affiliations:** 1 Department of Chemistry, University of Milan, Via Golgi 19, 20131 Milano, Italy; 2 Dipartimento di Scienza e Alta Tecnologia and To.Sca.Lab, Università Degli Studi dell’Insubria, 22100 Como, Italy; 3 ESRF, The European Synchrotron, 71, Avenue des Martyrs, CS40220, CEDEX 9, 38043 Grenoble, France; 4 Dipartimento di Scienze Della Terra “Ardito Desio”, University of Milan, Via Botticelli 23, 20133 Milano, Italy; 5 CNR-SCITEC, Via Golgi 19, 20133 Milano, Italy; 6 CNR—Istituto di Cristallografia and To.Sca.Lab, Via Valleggio 11, 22100 Como, Italy; 7 Department of Chemistry, University of Pavia, V.le Taramelli, 12, 27100 Pavia, Italy; 8 INSTM, Via Giusti 9, 50121 Florence, Italy

**Keywords:** ceria nanoparticles, total scattering, Debye Scattering Equation, Pair Distribution Function, atomistic simulations, ESR, Raman spectroscopy

## Abstract

We present a combined real and reciprocal space structural and microstructural characterization of CeO_2_ nanoparticles (NPs) exhibiting different crystallite sizes; ~3 nm CeO_2_ NPs were produced by an inverse micellae wet synthetic path and then annealed at different temperatures. X-ray total scattering data were analyzed by combining real-space-based Pair Distribution Function analysis and the reciprocal-space-based Debye Scattering Equation method with atomistic models. Subtle atomic-scale relaxations occur at the nanocrystal surface. The structural analysis was corroborated by ab initio DFT and force field calculations; micro-Raman and electron spin resonance added important insights to the NPs’ defective structure. The combination of the above techniques suggests a core-shell like structure of ultrasmall NPs. These exhibit an expanded outer shell having a defective fluorite structure, while the inner shell is similar to the bulk structure. The presence of partially reduced $O_{2}^{-\delta }$ species testifies to the high surface activity of the NPs. On increasing the annealing temperature, the particle dimensions increase, limiting disorder as a consequence of the progressive surface-to-volume ratio reduction.

## 1. Introduction

Investigating the structure of nanoparticles (NPs) is a difficult but essential task to fully rationalize their physical properties in the framework of the structure <-> physical properties paradigm of material science. To this purpose, we should better define what is meant by “structure” in the case of nanostructured materials. Traditional diffraction tools such as the Rietveld method [1] make use of the Bragg scattering to find the “average structure” with an accuracy that is limited by diffraction peaks broadening, especially in the case of very small and highly strained NPs. Deviations from the ideal structure, such as like finite-size defects and surface relaxations, which might significantly affect the NPs’ physical properties, also contribute to the diffuse scattering, which is neglected in the Rietveld analysis. Total scattering methods that make explicit use of both Bragg and diffuse scattering, such as the Pair Distribution Function (PDF) [2,3,4,5,6,7] and the Debye Scattering Equation (DSE) [8,9,10,11] approaches, are intrinsically suited to overcome this obstacle and supply accurate structural (and microstructural) findings for a deep comprehension of the physical properties of NPs. Though total scattering methods performed in reciprocal (DSE) or direct (PDF) space are in principle interchangeable (they contain the same information), differently optimized instrumental setup and data collection/reduction procedures make them complementary [12]. In this paper, we exploit such complementarity by applying the two methods to the intriguing case of ceria NPs. Cerium oxides exploit easy oxygen exchange [13,14] and charge transport performances [15]. While exchanging oxygen with the atmosphere, CeO_2−*δ*_ undergoes oxidation-reduction cycles, based on the Ce^4+^/Ce^3+^ redox couple, as expressed by the following defect equation [16,17,18]:

(1) $$2Ce_{Ce}+O_{O}⇌2Ce_{Ce}^{\prime }+V_{O}^{●●}+\frac{1}{2}O_{2}$$

For each oxygen vacancy formed, two electrons are injected into Ce_4f_ states [19,20], which diffuse through a small-polaron mechanism [16,21]. The oxygen vacancies $V_{O}^{●●}$ and Ce^3+^ ($Ce_{Ce}^{\prime }$) concentrations depend on temperature and oxygen partial pressure [17] and are boosted in nanocrystals due to the lowering of the heat of reduction [22,23]. Correlations between oxygen vacancies $V_{O}^{●●}$ and particle dimensions was pointed out by, e.g., Kung et al. [24]. The defect chemistry described by Equation (1) donates high oxygen storage capability to cerium oxide [25], which paves the way to the widespread use of CeO_2−*x*_ compounds in many different fields of catalysis, spanning from promoters in three-way catalysts (TWCs) for the emission control of auto-exhaust polluting gases [26], to hydrocarbon reforming [27], water gas shift reforming [28], selective hydrogenation [29], photocatalysis [30], and even biomedical applications [31]. 

Moving to the crystal structure, CeO_2−*δ*_ compounds maintain the very simple fluorite structure of CeO_2_ with space group $Fm\bar{3}m$, Ce in (0, 0, 0) and O in (1/4, 1/4, 1/4) sites. Redox processes occur in ceria, maintaining its structural integrity: the average crystal structure of CeO_2−*δ*_ remains fluorite in a wide range of oxygen non-stoichiometry. Reducing CeO_2_ down to CeO_~1.7–1.8_ leads to a disordered non-stoichiometric fluorite-related phase [32,33]. By further increasing the oxygen non-stoichiometry δ, a number of fluorite-related superstructures form, lowering the cell symmetry owing to vacancy orderings along the fluorite <111> direction. A comprehensive description of the superstructures in CeO_2−*δ*_ is given by a single crystal neutron diffraction study [34]. 

It turns out that the concentration and the ordering of the oxygen vacancies play a crucial role in defining the structure. In addition, the formation of an oxygen vacancy on ceria is surface sensitive, thus affecting the material redox and catalytic properties [25]. This prompted the investigation of defect structures at the atomic scale, focusing on nanoscale properties. One of the first applications of total scattering on undoped ceria demonstrated the presence of interstitial oxygen ions triggered by suitable annealing processes [35]. A more recent work still based on neutron PDF investigated defects on NPs of different shapes, suggesting the formation of surface oxygen defects consistent with reduced Ce_3_O_5+*δ*_ [36]. Concerning the first neighbor interactions, X-ray absorption spectroscopy revealed the contraction of the Ce–O atom pairs while decreasing the size of the ceria NPs [37], an effect opposite to the expansion observed by many authors [38,39,40], consistent with the larger ionic size of Ce^3+^ with respect to Ce^4+^ [41]. Such a contraction, however, has been explained in doped and undoped ceria as a result of the repulsion between O ions and O vacancies, which push the O ions towards the Ce ions from PDF [42,43] and molecular dynamics [44]. 

In this framework, the goal of this work is to investigate systematically the correlation between the local and the average crystal structure as a function of the crystal size of ceria NPs. To this purpose, ultrasmall CeO_2_ NPs (~3 nm) were synthetized with an inverse micellae method, while larger NPs were obtained by annealing thermally part of the original NP batch at different temperatures. The samples’ structures and microstructures were investigated by synchrotron radiation diffraction, combining conventional reciprocal space methods such as Rietveld refinements and Williamson-Hall (WH) analysis with total scattering methods based on the Debye Scattering Equation (DSE) and the Pair Distribution Function (PDF) in the reciprocal and direct space, respectively. The combined use of DSE and PDF (for the smallest NPs) allows an accurate picture of the structural relaxations at the NPs’ surface at the atomic level. As an additional insight of the analysis, the limits of the conventional diffraction methods to obtain the essential structural and microstructural features of ultrasmall NPs are evidenced. Raman and Electron Spin Resonance (ESR) spectroscopies complete the picture, evidencing the complex defect chemistry of CeO_2_ NPs and their high capability of storing active oxygen species at their surface. The experimental results are then integrated by ab initio and force field calculations to recognize the different contributions to structural relaxation in NPs.

The combination of all the above techniques suggests the formation of a core-shell-like structure in ultrasmall NPs, composed of a Ce^3+^ rich fluorite structure, with expanded/contracted interatomic shell/core distances, in comparison to the bulk structure. The larger the annealing temperature, the larger the NPs’ coherent domain size, mitigating the surface effects.

## 2. Materials and Methods

### 2.1. Synthesis

All reagents were purchased from Sigma-Aldrich and used without further purification: Cerium (III) nitrate hexahydrate (99%), sodium hydroxide, n-octane (98%), n-BuOH (≥99.5%), EtOH (95%), cetyltrimethyl ammonium bromide (CTAB, ≥97%). 

CeO_2_ NPs were produced by a reverse micellar synthesis [45]. Two microemulsions were prepared: the first one by mixing an aqueous solution of cerium nitrate (0.13 M, 50 mL) with a solution (SOL_A) containing n-octane (100 mL), CTAB (20 g) and n-BuOH (18.5 mL); the second one by mixing an aqueous solution of NaOH (0.7 M, 50 mL) and SOL_A. The two emulsions were mixed together and continuously stirred for 1 h. NPs were extracted by centrifugation at 6000 rpm for 45 min, then washed first with ethanol and after with water. They were dried overnight at 100 °C in air and finally, after grinding with an agate mortar, calcined at 200 °C in air for 4 h, producing sample Ce200. Aliquots of the latter were calcined at different temperatures (T_ann_): 400 °C, 500 °C or 700 °C for 4 h (samples Ce400, Ce500 and Ce700, respectively). Another aliquot was fired at 900 °C for 72 h (sample Ce900).

### 2.2. X-ray Powder Diffraction Measurements

X-ray powder diffraction (XRPD) patterns were collected at the European Synchrotron (ESRF) in Grenoble, France, and at the Swiss Light Source (SLS, Paul Scherrer Institute) in Villigen, Switzerland. 

Data for Rietveld, line profile and PDF analysis were collected at 90 K at the High Resolution Powder Diffraction beamline ID22 of the ESRF, Grenoble [46]. The powders were filled into kapton© capillaries, aligned on the axis of the diffractometer and rotated to increase statistical orientations. For *Q*-space investigation, the high-resolution setup was adopted, collecting data in the 0 < 2θ ≤ 40° interval (*Q*_max_ = 12.6 Å^−1^) using the crystal analyzers on the diffracted beam [47] with incident wavelength λ = 0.354264(3) Å, defined with a Si NIST 640c standard. Each acquisition lasted 30 min, with longer counting times at high angles (20 ≤ 2θ ≤ 40°) to increase the signal to noise ratio. The size and strain analysis of the samples was carried out using the Williamson–Hall (WH) method [48]. Structural Rietveld refinements were performed via the GSAS software [49] and its graphical interface EXPGUI [50]. Data for PDF analysis were collected on Ce200, Ce500 and Ce900 samples at λ = 0.17125 Å (about 30 min/pattern) using a Perkin Elmer XRD 1611CP3 detector. 

An aliquot of the Ce200 sample was filled into a quartz capillary and measured at the ID15A beamline [51] of the ESRF (λ = 0.182329 Å), while heating at 4 °C/min rate from 200 to 820 °C. The frames, collected with a Pilatus3 X CdTe 2 M two-dimensional detector, were grouped to obtain patterns spanning 2.5 min and 10 °C ranges each. Empty capillaries were measured at 90 K and RT, respectively, adopting the same beamline configurations as for the samples, but longer counting times, in order to subtract their scattering signal from each specimen experimental curve and to retrieve only the diffraction signal coming from the sample. 2D images were integrated using pyFAI [52]. PDF data up to *Q*_max_ = 28 Å^−1^ (ID22) and 24 Å^−1^ (ID15A) were reduced using pdfgetX3 [53]. Real-space refinements were carried out by PDFgui [54].

The PDF is here described using G(r) formalism, which reflects the probability of finding a pair of atoms separated by a distance *r* with an integrated intensity dependent on the pair multiplicity and the scattering factors of the elements involved. G(*r*) is experimentally determined via sine Fourier transform of the total scattering function F(*Q*), which corresponds to the coherent scattering coming from the sample (Bragg peaks and diffuse scattering) after proper normalization [4]:

(2) $$G\left(r\right)=\frac{2}{\pi }\overset{\infty }{\underset{0}{\int }}F\left(Q\right)\sin\left(Qr\right)dQ$$

Room temperature total scattering measurements on Ce200 and Ce500 samples for DSE analysis were performed at the MS-X04SA beamline of the SLS (Paul Scherrer Institute) [55]. A beam energy of 22 keV was set, and the operational wavelength (0.56363 Å) was accurately determined using a silicon powder standard (NIST 640d). Data were collected in the 0.4–130° 2θ range using a single-photon counting silicon microstrip detector (MYTHEN II) [56]. Scattering from the sample holder and from the empty glass capillary were independently collected under the same experimental conditions. Angle-dependent intensity corrections were applied to the raw data to account for sample attenuation due to absorption effects; sample absorption curves were determined using an X-ray tracing method [57] and by measuring the transmitted beam from the filled capillary at room temperature, while for the empty capillary the X-ray attenuation coefficient was computed using its nominal composition. Angular calibrations were applied to the zero angle and x, y capillary offsets, derived from the certified silicon powder standard (NIST 640 d) using locally developed procedures. Background and (absorption-corrected) capillary scattering contributions were subtracted from the sample signal. Some insights on the DSE modelling approach and on the models adopted for data analysis are reported in the Supplementary Materials.

### 2.3. Electron Spin-Resonance and Micro-Raman Spectroscopy Measurements

ESR spectra were collected by a Bruker ELEXSYS spectrometer equipped with an ER4102ST standard rectangular cavity at X band (9.4 GHz) frequency at room temperature. The derivative dP/dH of power P absorbed was recorded as a function of the static magnetic field H. Selected features of the experimental spectra were fitted using Easyspin software package [58] implemented in the MATLAB environment [59].

Micro-Raman spectroscopy was carried out using a Horiba LabRam HR evolution at the Dipartimento di Scienze della Terra “A. Desio” of the University of Milan. The spectrometer was equipped with Nd-YAG 532 nm/100 mW with Ultra Low Frequency filters. Scattered light was collected by a 100× objective (numerical aperture = 0.9) in backscattering geometry; a diffraction grating with 600 lines/mm and the hole set at 100 μm were used. The spectrum was detected by a Peltier-cooled CCD. To balance signal to noise, two accumulations for 20 s were collected. Instrument calibration was performed before each round of analysis using the peak at 520.70 cm^−1^ of a silicon wafer.

### 2.4. Atomistic Simulations

Atomistic simulations of ceria NPs were performed with a multiscale approach. First, the surface energy of the low index hydroxylated ceria surfaces was determined using Density Functional Theory (DFT) calculations, exploiting the plane wave code Quantum Espresso [60,61] and the PBE functional, ultrasoft pseudopotentials, including *f* electrons in valence [62] with a cutoff of 50 Ry. Slab models of the surface were constructed with a minimum of 8 atomic layers, adding –OH groups to the surface Ce atoms and –H to the surface O atoms. The structures were fully relaxed, and the surface energy was determined by subtracting the energy of the equivalent number of bulk unit formulas and the energy of an equivalent number of water molecules. The calculated energies of the hydroxylated {100}, {110} and [111} surfaces were 0.63, 0.33 and 0.97 J/m^2^, respectively.

Next, large atomistic models of the NPs were constructed using the Wulff construction based on the DFT calculated surface energies, using the MPinterfaces code [63] to construct NPs with the largest linear dimension up to 40 Å, terminated by dry surfaces. Then, –OH and –H groups were added to the surface atoms in order to simulate NPs grown in wet conditions.

Finally, an ab initio derived reactive force field (ReaxFF) [64,65] was used to simulate dry ceria nanoparticles. To account for the hydrogen atoms, the O··H interaction from the ReaxFF was used to describe iron oxyhydroxide [66]. The mixing of force field parameters is justified by the fact that H is always bound to an oxygen, and the Ce··H interaction is negligible. The geometry of the wet NPs was relaxed with the LAMMPS code [67].

## 3. Results

### 3.1. X-ray Powder Diffraction: Bragg-Scattering Analysis by Rietveld and Williamson-Hall (WH) Methods

We start our investigation by applying two typical tools for structural and microstructural study, namely the Rietveld refinements and the WH analysis, to high resolution patterns. 

**Figure 1:**
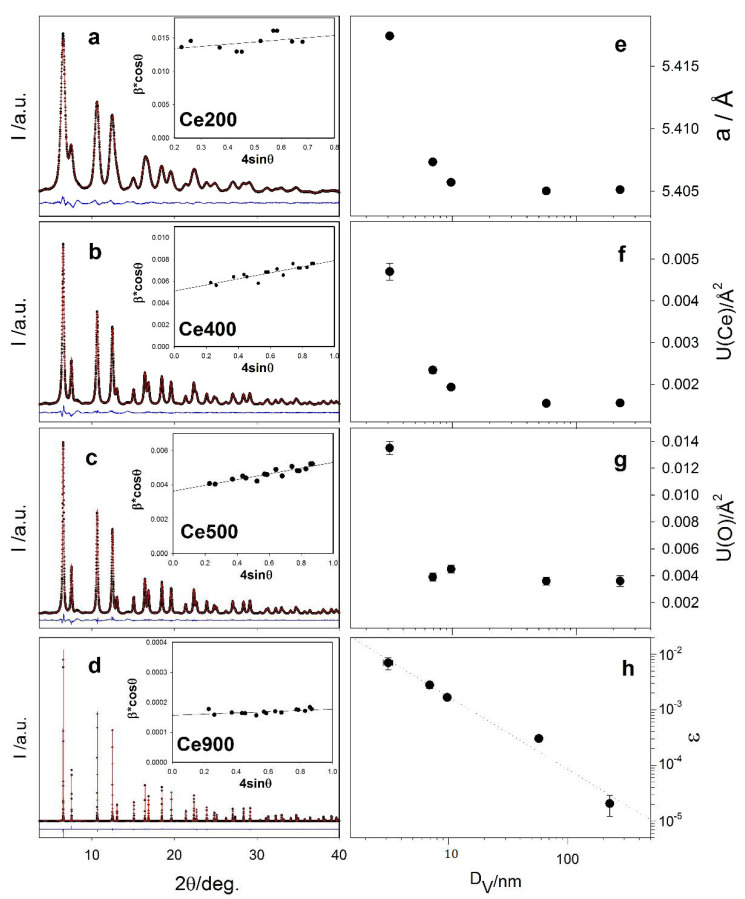
Experimental XRPD patterns (black dots), best fits of Rietveld analysis (red curves) and residuals (blue curves) collected on samples Ce200, Ce400, Ce500 and Ce900 (panels (**a**–**d**)). The WH analysis of the same patterns is included in the insets. Selected refined parameters of the Rietveld refinements are reported in panels (**e**) (cell parameter) and (**f**,**g**) (Ce and O displacement parameters) as a function of the crystallite dimension D_V_. Panel (**h**) shows the trend of strain versus D_V_ according to WH analysis results.

In Figure 1, the experimental XRPD patterns measured at 90 K on Ce200 (a), Ce400 (b), Ce500 (c) and Ce900 (d) are shown as black crosses. Bragg peaks broaden on lowering the annealing temperature indicating a progressive decrease of coherence length mainly due to reduced crystallite dimensions and/or increased microstrain contributions. To quantify the two effects, we adopted the WH model [48]. After subtracting the small instrumental contribution to broadening using a LaB_6_ standard, the peaks’ integral breaths *β* are plotted as a function of their position according to the formula: $\beta \cdot cos\theta =4\varepsilon sin\theta +\lambda /D_{V}$, where $D_{V}$ is the volume-weighted crystallite dimension and ε is the microstrain parameter. The WH plots are reported in the insets of panels (a–d) of Figure 1; $D_{V}$ and ε are reported in Table 1. The adopted synthesis and low annealing temperatures produce highly strained (ε almost 10^−2^) and very small NPs ($D_{V}$ ≈ 3 nm). At higher T_ann_, nanocrystals grow by almost two orders of magnitude, while ε reaches values as small as ε ≈ 2 × 10^−5^, typical of low-defect well-grown phases.

The patterns were fitted against the fluorite model (space group $Fm\bar{3}m$) using the Rietveld method. No evidence of superstructure peaks or signal of oxygen vacancy ordering [68] were observed. Some diffraction signals of patterns reported in Figure 1 (e.g., those at ≈ 4 and ≈ 8 deg.) are not correctly interpreted by the fluorite structural models. These signals, not observed on the same specimens collected on other instruments, are instrumental parasitic scattering, likely arising from a crosstalking effect [47]. 

During the fitting procedure, the cell constant *a* and the isotropic atomic mean square displacements *U*(Ce) and *U*(O) were relaxed. Figure 1a–d reports the best fits as red curves, while the fitted parameters are shown in Table 1. Some misfits evident at low angle values in Figure 1a–c will be discussed in the following section. Parameters *a*, *U*(Ce) and *U*(O) are displayed in panels e–g of Figure 1, respectively, as a function of the crystallite dimension *D*_V_ estimated by WH. The largest particles present cell parameters and displacement parameters in line with previous investigations [42,43,68,69]. While the crystallographic coherence domain becomes smaller, all the parameters relax to higher values: the cell constant grows by about 0.2% for specimen Ce200, characterized by very small NPs (~ 3 nm). This is a well-known nano-structuring effect on oxides in general [39] and nano-CeO_2_ in particular [40], which, in the latter case, is attributed either to a significant concentration of Ce^3+^ species or to surface structure relaxation [70]. The concomitant increase of the displacement parameters suggests structural disordering in small NPs; with the patterns collected at the same temperature, the increase of *U*(Ce) and *U*(O) is attributed to a static contribution to the atomic mean square displacement parameters [71,72], possibly due to surface phenomena. 

In Figure 1h the ε vs. *D*_V_ plot is shown in log-log format. The data are adequately fitted by a straight line (dotted line) with slope = −1.3(1). Since the surface to bulk ratio is proportional to $D_{V}^{-1}$, we suppose that strain effects are mainly due to surface properties. 

Summarizing, Rietveld and WH analyses point to unit cell expansion and increased structural and microstructural disorder in small NPs. Aiming to map accurately and quantitatively the structural reconstruction appearing in CeO_2_ NPs, we move to total scattering methods.

### 3.2. Total Scattering Analysis

#### 3.2.1. Q-Space Analysis Using Debye Scattering Equation

**Figure 2:**
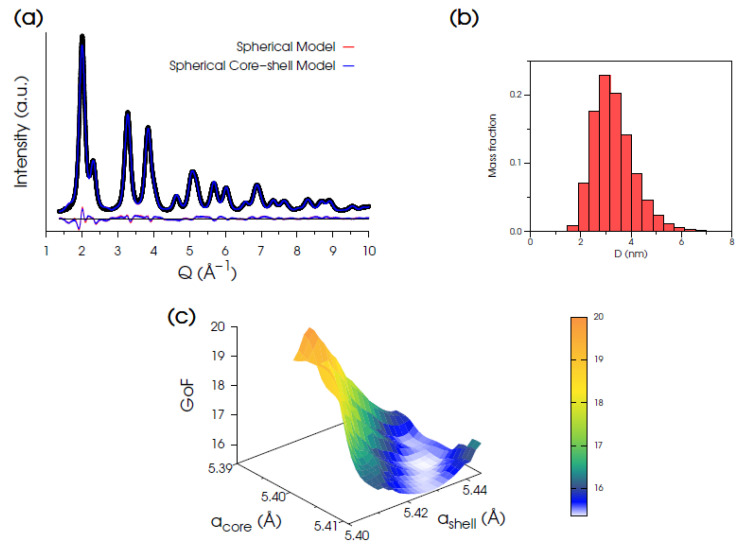
(**a**) Synchrotron XRPD data (black dots) of Ce200 and DSE best fits obtained using either a uniform spherical model (red trace, GoF = 15.54) of CeO_2_ NPs or an optimized homo core-shell spherical one (blue line, GoF = 14.99) with diverse lattice parameters in the core and the in shell. (**b**) Histograms of the refined monovariate mass-based lognormal size distribution function of NCs. (**c**) 3D hypersurface of GoF versus *a*_core_ and *a*_shell_ explored for the optimization of the homo core-shell spherical model.

Total scattering data of Ce200 were first modeled using a fluorite structure and assuming a spherical nanoparticle morphology, as detailed in the Supplementary Materials. For this model, the best pattern fit (Figure 2a, red line), with statistical indicators *R_wp_* = 6.14% and *GoF* = 15.54 (being $GoF={\left(\chi^{2}\right)}^{1/2}$), provides a lattice parameter *a* = 5.4155 Å and mass-based average diameter <D>_M_ = 3.31 nm with relative distribution σ/<D>_M_ = 0.24 (from the refined lognormal size function shown in Figure 2b). While the high-*Q* region (4.5–15.8 Å^−1^) is well described by this model, the low-*Q* match (≈0.5–4.2 Å^−1^) remains quite unsatisfactory, as noticeable in the residuals of Figure 2a (red line). This behavior suggests a non-uniform lattice periodicity within the nanocrystal volume and points to the occurrence of a surface relaxation [73] or, in other words, to a homo-core-shell structure in which the shell lattice parameter is expanded with respect to that in the core. Such a hypothesis is also in agreement with previous reports on nanoceria having a Ce^3+^ enriched surface layer [40,74,75]. Results are summarized in Table 2. Accordingly, a spherical core-shell model was developed, consisting of either a single- (≈0.42 nm thick) or a bi-layer lattice node shell surrounding the core.

In order to determine the optimized *a_core_*/*a_shell_* pairs, a grid search exploration was performed using both the single- and bi-layer models. The 3D hypersurface for the single-layer model is shown in Figure 2c. The atomistic model providing the best fit (the minimum of the 3D map) is obtained at *a_core_* = 5.405 Å and *a_shell_* = 5.436 Å, with a relative shell-to-core expansion of 0.57%, and nearly unchanged size parameters (<D>_M_ = 3.34 nm, and σ/<D>_M_ = 0.30, Figure 2b). The statistical indicators (*R_wp_* = 5.92%, GoF = 14.99) suggest a slight improvement compared to the uniform spherical model (Figure 2a, blue line). Indeed, the peak position in the middle-*Q* region is almost fully recovered, and the high-*Q* region is still well-matched.

Nonetheless, the persistence of a misfit of the position and intensity of the 111 peak suggests that some additional structural/microstructural effects need to be considered. Inspired by the anisotropic morphology reported for CeO_2_ particles grown as nanorods in alkaline conditions [76] that expose preferentially {110} and {100} facets, we analyzed the influence of a similar morphology in combination with appropriate faceting on the Ce200 DSE-fit.

Different models with prismatic morphology and NPs grown along two independent directions were developed, which are synoptically collected in Figure S1 (details on the atomistic model construction are given in the Supplementary Materials).

The model adopting a tetragonal I-centered lattice with unit cell vectors **a**_t_ = **a**_k_, **b**_t_ = (**b**_k_ + **c**_k_)/2 and **c**_t_ = (−**b**_k_ + **c**_k_)/2 (modulus *b*_t_ = *c*_t_ = √2/2 × *a*_k_) was the most promising. Prismatic NPs were grown along the directions parallel to **a**_t_ and **c**_t_, and both uniform and core-shell models with fixed shell thickness were tested. In all cases, NP sizes were refined according to a bivariate lognormal distribution function [77].

**Figure 3:**
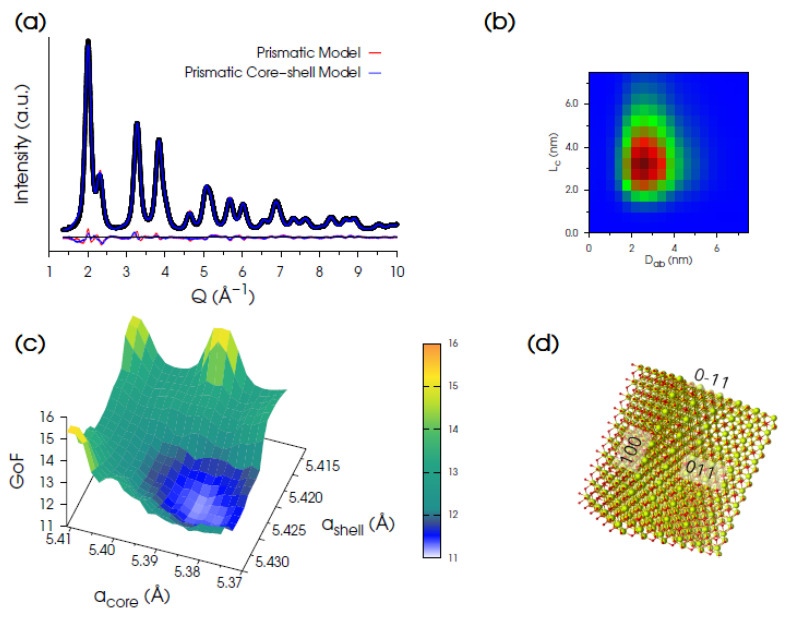
(**a**) Synchrotron XRPD data (black dots) of Ce200 and DSE best fits obtained using either a prismatic model (shown in panel (**d**)) with a uniform unit cell parameter (red trace, GoF = 12.35) or an optimized homo core-shell prismatic model (blue line, GoF = 10.94). (**b**) 2D map of the refined bivariate lognormal size distribution function of NCs in the L_c_ and D_ab_ coordinates (D_ab_ is the diameter of equivalent volume to the prism basal plane). (**c**) 3D hypersurface of GoF versus *a*_core_ and *a*_shell_ explored for the optimization of the homo core-shell prismatic model. (**d**) Prismatic model of CeO_2_ NCs exposing {011} and {100} facets.

The best fit (Figure 3a) was obtained using a bi-layer prismatic core-shell model exposing {011} and {100} facets (in cubic notation, Figure 3d), providing mass-averaged sizes: <L_a_>_M_ = 3.26 nm, <L_b_>_M_ = 2.31 nm, and <L_c_>_M_ = 3.28 nm and relative dispersion σ/<L_a_>_M_ = σ/<L_b_>_M_ = 0.31; σ/<L_c_>_M_ = 0.41 (Figure 3b). The best *a_core_* = 5.386 Å and *a_shell_* = 5.424 Å lattice parameters were achieved, once more using a grid search algorithm (the 3D hypersurface is shown in Figure 3c). The final match of the calculated vs. experimental pattern is largely improved by this model, as witnessed by the significantly lower statistical indicators (*R_wp_* = 4.32%, GoF = 10.94) favoring the core-shell structure vs. the uniform prismatic one of identical morphology/faceting of NPs (GoF = 12.35). The relative shell-to-core expansion is 0.72%. This finding is in agreement with the presence of Ce^3+^ species preferentially located at the NP surface, in line with previous reports [40,74,75].

Moving to higher T_ann_, the DSE-based analysis of the Ce500C sample provided minor difference in the agreement factors of the prismatic vs. spherical models (GoF = 12.09 vs. 12.67, respectively) and suggested a fluorite type structure and a uniform unit cell parameter *a* = 5.4112 Å throughout the NP volume, as a consequence of the reduced surface-to-volume ratio of larger NPs in the sample.

The mass-based average sizes are <L_a_>_M_ = 9.85 nm, <L_b_>_M_ = 6.97 nm, and <L_c_>_M_ = 12.72 nm (σ/<L_a_>_M_ = σ/<L_b_>_M_ = 0.30; σ/<L_c_>_M_ = 0.23). The experimental data and the DSE best fit for this sample are reported in Figure S2.

#### 3.2.2. r-Space Analysis by Pair Distribution Function

**Figure 4:**
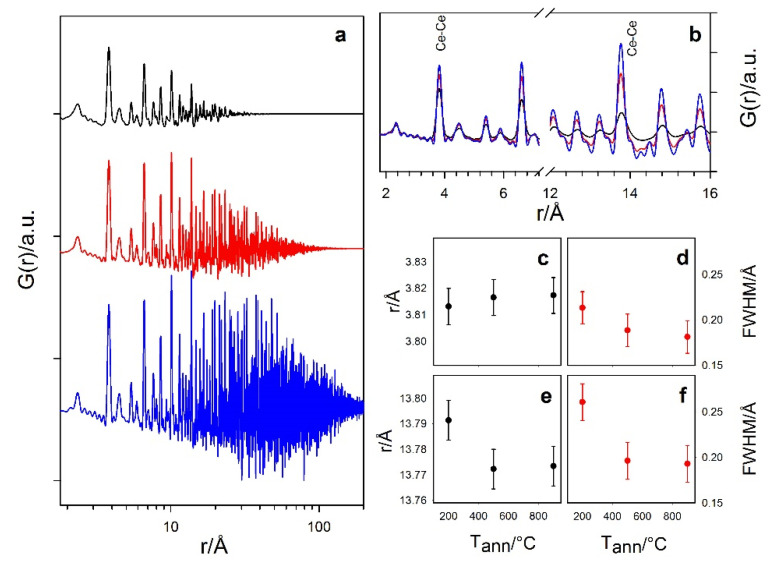
(**a**): Experimental G(*r*) curves of Ce200, Ce500 and Ce900 samples, from top to bottom, shifted along the y axis for clarity. (**b**) Same curves superposed and zoomed in two intervals. Labels identify two Ce-Ce peaks for single peak fitting with a Gaussian function at ~3.8 Å and 13.8 Å. The peak center and FWHM are reported in panels (**c**–**f**).

Total scattering data of Ce200 were first modeled using a fluorite structure and assuming spherical NPs. Figure 4 shows the experimental G(*r*) curves of Ce200, Ce500 and Ce900 samples as black crosses. The lower the annealing temperature, the shorter the *r* range with non-negligible G(*r*) peak amplitude, as expected for small NPs. These G(*r*) were analyzed using direct analysis and real-space Rietveld-based modeling.

Zooming into the short-range part of the curves and focusing on some of the most intense Ce–Ce pairs (e.g., the ones labelled as Ce–Ce in Figure 4), we observe that peaks broaden when reducing the particle dimension. Indeed, since all the G(*r*) have been collected at the same temperature and using the same experimental setup, this effect has to be attributed to static disorder, such as surface-induced lattice relaxation [78]. In addition, as quantified by the direct analysis of the peaks using single Gaussian functions, while the peaks of different samples are centered at almost the same interatomic distance at low-*r*, extending the *r* range brings a shift of the peaks of Ce200 toward larger values. In Figure 4c–f, the positions and the FWHM values for Ce–Ce distances around 3.8 and 13.8 Å are reported as examples.

The same G(*r*) curves were analyzed using the real-space Rietveld method, by applying a fluorite structural model. The finite particle size was modelled using spherical particles as implemented in DiffPy [79] against experimental G(*r*) data in the 2–200 Å *r* range and allowing the D_V_ parameter to vary.

The refined parameters were D_V_ = 2.9(1), 9.9(2) and ≈10^4^ nm for Ce200, Ce500 and Ce900, respectively. The first two values match those from WH analysis. The too-large crystal size obtained for Ce900, in respect to the WH analysis of high resolution diffraction data, is attributed to the not-optimal instrumental resolution of the 2D setup: XRPD peaks of the Ce900 sample are almost as sharp as the ones of the silicon powder standard, making it difficult to separate the physical size contribution from that of the instrument. In addition, the trends of the cell parameter *a* and of the displacement parameters *U*(Ce) and *U*(O) on raising *T_ann_* are in accordance with the reciprocal space analysis: the Ce200 sample displays the largest values of all the parameters.

**Figure 5:**
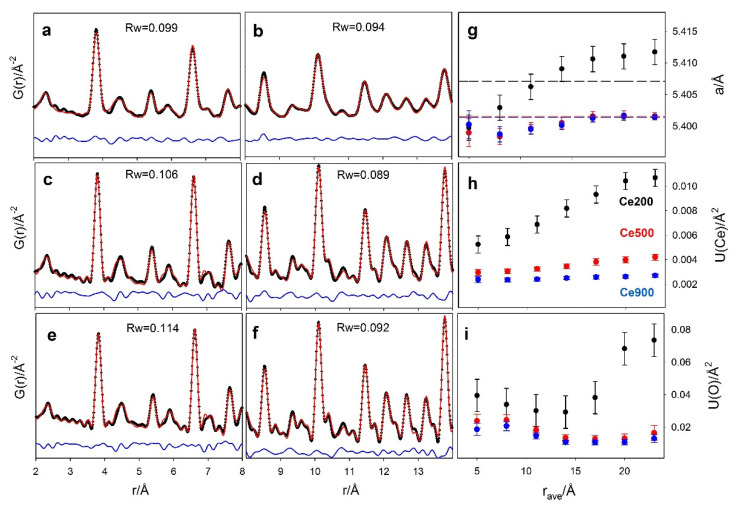
Real-space Rietveld refinements of G(*r*) collected on Ce200 (**a**,**b**); Ce500 (**c**,**d**); Ce900 (**e**,**f**) in two different *r* ranges (2–8 Å and 8–14 Å). Experimental data, best fit and residuals are black crosses and red and blue solid lines, respectively. R_w_ values are also reported in each panel. Selected refined parameters of the real-space refinements are reported in panels (**g**) (cell constants a) (**h**,**i**) (Ce and O displacement parameters) as a function of the centroid of the *r* range of the box-car refinements. Refined parameters from measurements on the Ce200, Ce500 and Ce900 samples are reported as black, red and blue circles, respectively.

The real-space analysis allows mapping the evolution of the same structural parameter varying the investigated *r* range, using a box-car approach, i.e., by applying a structural model progressively in different *r* ranges, to monitor possible structural evolutions [68,78,80]. The fluorite structural model was applied to all three datasets using 6 Å wide *r* ranges from 2 Å up to 26 Å. For larger *r* ranges, the G(*r*) amplitude for the Ce200 case is negligible. Figure 5 reports the refined parameters for Ce200 (black circles), Ce500 (red circles) and Ce900 (blue circles) against the centroid of the *r* intervals used in each refinement. The *r* evolution of the *a* cell constant has to be considered a measure of the structural relaxations at different coherence lengths. For ultrasmall NPs, it can be seen as an indicator of changes while progressively selecting the pair distances at the NP surface. At low *r*, the cell constants of all the samples are very close to each other (a ≈ 5.40 Å). On raising *r*, *a* increases to 5.41 Å for Ce200, while much smaller changes are observed for the remaining samples.

Let us consider the core-shell model suggested by DSE analysis, with larger interatomic distances in the shell with respect to the core. At the shortest *r* values, the interatomic distances sampled by PDF (averaged over the entire NP volume) are mainly core-core and shell-shell. However, while the atoms in the core have full coordination, as in the bulk fluorite, atoms at the surface, belonging to the shell, suffer from structure truncation. As a consequence, the average cell parameter is contributed mainly by the core rather than by the shell. When increasing the *r* interval value, the frequency of core-shell pairs increases, since the core atoms experience reduced long-range coordination. This causes the increase of the cell parameter observed in Figure 5. Eventually, when the *r* interval approaches the average nanoparticle size, the G(*r*) samples interatomic distances belonging to couples of shell atoms at opposite faces of the NPs. For this reason, *a* takes values larger than the average ones refined in the whole *r* range (see dashed black line in Figure 5).

**Figure 6:**
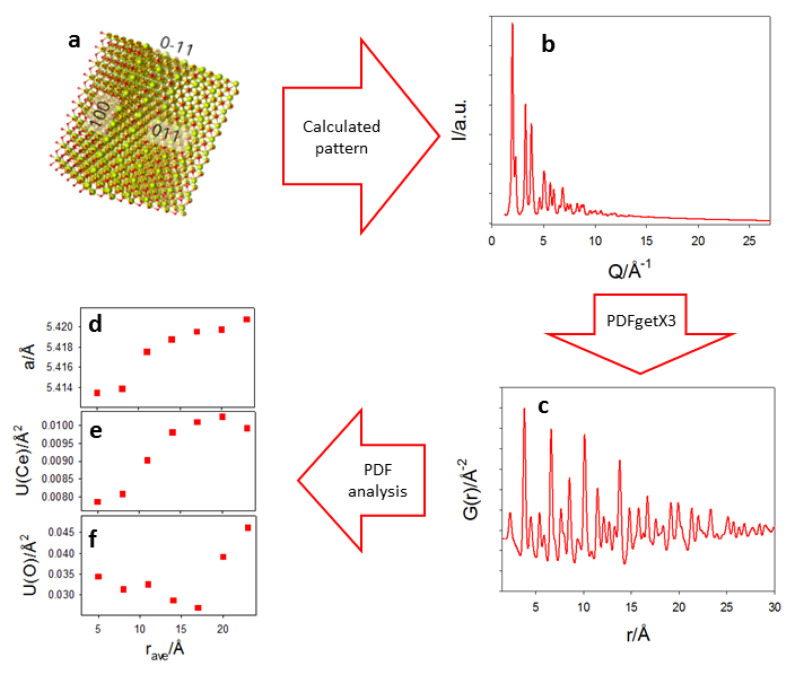
The prismatic core-shell model refined by DSE (**a**) produced a calculated pattern (**b**) that was normalized and Fast Fourier Transformed to obtain the PDF function displayed in (**c**). The G(*r*) was fitted using a box-car strategy. Refined cell parameter, U(Ce) and U(O) are displayed in (**d**–**f**).

To prove the consistency of the trend of the cell parameter as a function of *r* revealed by PDF analysis with the DSE results, we used the prismatic core-shell model refined by DSE (Figure 6a) to obtain a calculated pattern at the same *Q*-resolution of PDF experimental data (Figure 6b). This was normalized and Fast Fourier Transformed (FFT) using PDFgetX3 to obtain G(*r*) (Figure 6c). The obtained G(*r*) was then fitted using the same box-car strategy described above, obtaining the same growing trend of the cell parameter on increasing the interatomic distances revealed for the Ce200 sample (Figure 6d).

The displacement parameters *U*(Ce) and *U*(O) are shown in Figure 5h–i. Ce200 exhibits the largest values even at low *r*, confirming the increased positional disorder in small NPs. Moving to larger interatomic distances for the same sample, *U*(Ce) almost doubles, again in agreement with a core-shell model: different equilibrium interatomic distances exist in the inner/outer parts of the NPs, and an additional contribution to their distribution appears while passing from core-core/shell-shell dominated to core-shell dominated interatomic distances. Again, the box-car refinement results of the G(*r*) calculated by the DSE model (Figure 6e–f) show the same trends, in line with larger displacement parameters determined for Ce and O shell atoms in the core-shell model (see Table 2). As a final comment, only tiny changes of the refined parameters in different *r* ranges are observed in Ce500 and Ce900 samples, due their smaller surface to bulk volume ratio.

**Figure 7:**
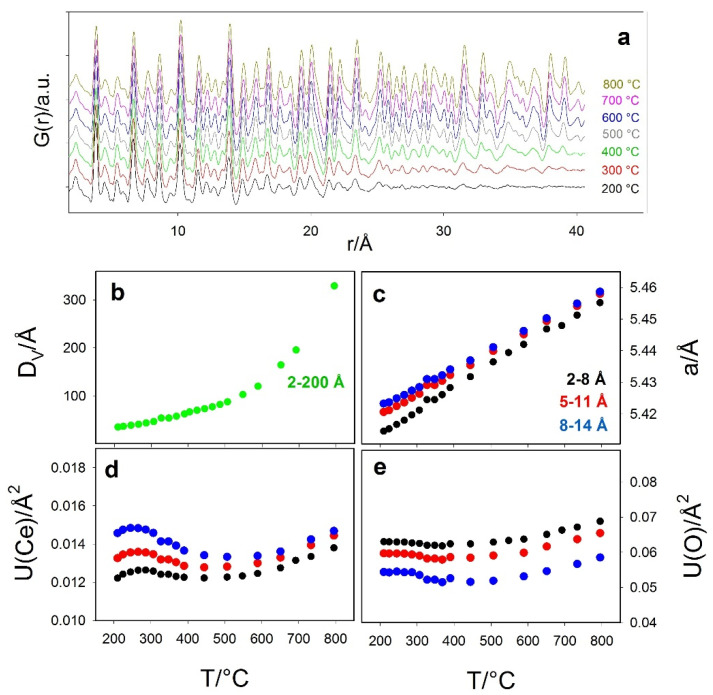
(**a**) Selected experimental G(*r*) collected in situ while heating Ce200 sample at 10 °C/min. (**b**–**e**) selected refined parameters as a function of T: (**b**) particle diameter, (**c**) cell constant, (**d**) displacement parameter Ce and (**e**) O. Black, red, blue and green circles refer to refinements in the 2–8 Å, 5–11 Å, 8–14 Å and 2–200 Å *r* intervals, respectively.

Since specimen Ce200 revealed the most interesting structural effects, it was subjected to a further in situ investigation while heating from 200 to 800 °C. Selected experimental G(*r*) are displayed in Figure 7.

Data were analyzed in the 2–150 Å range to extract the particle diameter D_V_ (see panel b), which was fixed during the refinements in the 2–8 Å, 5–11 Å and 8–14 Å intervals. Selected refined parameters are shown in panels c–e of Figure 7 as black, red and blue circles, respectively.

The particle dimensions increase on heating, with a rising slope above 500 °C. Again, the D_V_ values at 200 °C (3.4 nm) and 500 °C (8.9 nm) match with the previous determinations. The box-car refinement confirms the trends of *a*, *U*(Ce) and *U*(O) of the ex situ low *T* investigation; they increase at larger *r* intervals, and their differences become smaller at high temperature, as a consequence of the reduced surface to bulk ratio.

*U*(Ce) and *U*(O) display complex trends: at *T* < 400–500 °C, their curve exhibits a negative slope. Indeed, the refined values are contributed by atomic vibration and static disorder [71,72]: while the former term should increase on heating, the latter decreases upon nanoparticle growing, thus reducing the surface to bulk ratio. This is again evidence that disorder is mainly localized at the surface. For *T* > 500 °C, the surface volume fraction is small, and it hardly contributes to XRD broadening and to the damping of G(*r*) curves: the expected positive slopes for the displacement parameters are recovered.

### 3.3. Spectroscopy

#### 3.3.1. Electron Spin Resonance

**Figure 8:**
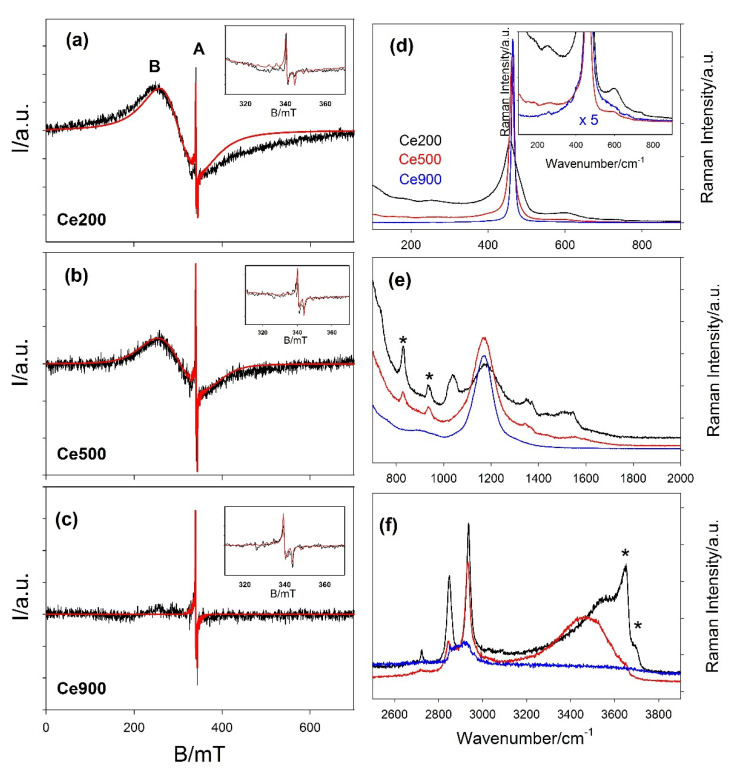
(**a**–**c**) Experimental ESR spectra collected at room temperature on the Ce200 (top), Ce500 (middle) and Ce900 (bottom) are reported as black curves. Features A and B are described in the main text. The detail of the A spectrum is reported in the insets. Spectral simulations realized by the model described in the main text are represented by red curves. (**d**–**f**) Raman spectra of Ce200 (black), Ce500 (red) and Ce900 (blue) in the (**d**) 200–900 cm^−1^, (**e**) 700–1900 cm^−1^ and (**f**) 2500–4000 cm^−1^ ranges. The insets highlight the less intense features of the same spectra. To this purpose, the blue curve has been multiplied by 5.

Figure 8 reports the ESR spectra collected at room temperature on the Ce200, Ce500 and Ce900 samples as black curves. The spectrum of Ce200 (upper panel) presents two features labeled A and B. Feature A (highlighted in the inset) consists of a sharp axial ESR signal characterized by $g_{⊥}\approx 1.97$, $g_{∥}\approx 1.95$ and $\Delta H_{PP}\approx$ 0.5 mT. Label B is placed on the maximum of a broad ($\Delta H_{PP}\approx$ 90 mT) ESR peak. The best simulation of this feature is obtained introducing a slightly axially distorted symmetry ($g_{⊥}\approx 2.21$, $g_{∥}\approx 2.24$). The simulated spectra are reported as red curves in Figure 8.

Axial ESR peaks like A are often reported for nanostructured CeO_2_. Several authors have attributed it to the presence of paramagnetic Ce^3+^ ions [81,82,83,84]. Conversely, Figaj and colleagues [85] questioned this interpretation, noting that ESR signals of 4f^1^ ions should not be detected above 20 K because of the strong spin-orbit coupling [86]. They attributed feature A to impurities such as chromium ($g_{⊥}$= 1.964 and $g_{∥}$= 1.943) or gadolinium ($g_{⊥}$= 1.975 and $g_{∥}$= 1.950). In particular, the g and $\Delta H_{PP}$ values experimentally determined in this study are compatible with ca. 0.01% Gd or 0.005% Cr low doping levels, according to the analysis of the progressive ESR line broadening on Gd doping of CeO_2_ performed by de Biasi and Grillo [87] and by Figaj and colleagues [84], respectively, with the two ions. Furthermore, the above-reported Gd doping value would be compatible with the possible presence of Gd impurity in the precursor declared by the supplier of the cerium nitrate. Table 3 reports the refined parameters using the gadolinium to fit feature A. Nevertheless, we cannot exclude a priori the possible presence of chromium in trace. As a consequence, we fitted the same spectra using this instead of Gd^3+^ in the model. The resulting parameters are reported in Table S1. In any case, the attribution of this ESR line is beyond the scope of the present paper. We note that feature A remains almost constant for all the spectra, as reported in Table 3. Should surface Ce^3+^ be at the origin of feature A, its concentration would be expected to vary with the particles’ dimensions. Since feature A remains almost constant for all the spectra, the simulated spectrum of the Ce200 sample is calculated supposing the presence of impurities. The very low impurity concentration seems to be the same in all the samples; thus, it should not affect the changes of the structure and of the physical properties on varying the annealing temperature.

Passing from Ce200 to Ce500, feature B reduces its intensity compared to feature A and almost disappears in the case of Ce900. For this reason, the parameters used to simulate it in the spectrum of the Ce900 sample have to be considered in a qualitative manner. Several authors have pointed out the presence of superoxide surface species $O_{2}^{-}$ featuring g values larger than two [81]. Xu and colleagues [88] attributed the formation of superoxide species on a reduced ceria surface to the activation of molecular oxygen from air by Ce^3+^, forming $Ce^{4+}-O_{2}^{-}$ species. Due to the high concentration of superoxide radical ions, the peak broadens significantly, presumably due to large electron–electron interactions at higher coverage, hence concomitantly decreasing electron relaxation times, as proposed in [88]. The presence of $O_{2}^{-}$ species is in accordance with the exposure of the (110) surface of the Ce200 sample revealed by DSE [89]. While for samples where CeO_2_ exposes almost exclusively the (111) surface, Raman fingerprints of superoxide appear only below 213 K [90]; in NPs exposing the (100) surface they are observed up to 470 K [91]. Feature B evolves on raising T_ann_ (and D_V_), reducing its intensity as expected, for the increased volume-to-surface ratio, which reduces the concentration of O_2_^−^ species in the sample.

#### 3.3.2. Raman Spectroscopy

Raman spectroscopy is widely adopted to reveal the symmetry breaks in CeO_2_ compounds [92,93,94,95]. Theoretical and computational work allows rationalizing the experimental findings [93,96,97]. A recent review summarized much of the theoretical and experimental Raman findings on CeO_2_ compounds even under operating conditions [98].

Raman spectra of Ce200, Ce500 and Ce900 samples are displayed in Figure 8d–f as black, red and blue curves, respectively. Panel d reports data in the 100–900 cm^−1^ interval. The spectrum of the Ce900 sample is dominated by a sharp peak at 464.6 cm^−1^ that corresponds to the only active first order Raman optical phonon of fluorite CeO_2_ with triple degenerate F_2g_ symmetry [92,93,96,97]. It is in general viewed as the symmetric breathing mode of the O atoms around each cation [92], but it involves both the Ce-O and O-O force constants [98,99]. Moving to lower T_ann_ and smaller particle dimensions, the F_2g_ peak is red and shifted to 461.3 and 456.1 cm^−1^ for samples Ce500 and Ce200, respectively. Moreover, it broadens and becomes asymmetric, and a tail appears at the low energy side in Ce200. Such nanostructuring-related effects were observed in CeO_2_ by several authors [93,95]. Nanostructuring also promotes the growth of signals at both sides of the main peak. To highlight them in the inset, the y axis was expanded, and the spectrum of the Ce900 was multiplied by a factor of five. All spectra present a peak around 250 cm^−1^, which is attributed to surface Ce-OH vibrations [94]. Conversely, Shilling et al. attributed it (as well as the peak around 402 cm^−1^) to the surface termination of the NPs [93]. In both cases, the high surface to bulk ratio boosts its intensity. According to DFT calculations at 256 cm^−1^, a 2TA overtone of the CeO_2_ structure is present [93].

The feature(s) in the 500–600 cm^−1^ range (often called the D band) have been attributed by several authors to modes raising from the symmetry breaks originated by oxygen vacancy formation in both pure [93,95,98] and doped ceria [92,96,100,101]. The intensity of the D band is larger in the smallest nanocrystals (see panel d), and its shape suggests that it sums up several contributions. Indeed, DFT calculations showed that the mode energies depend on the valence of the involved Ce and the presence/absence of an oxygen vacancy in its cubic cage [93], with the largest values being attributed to Ce^4+^O_7_V^●●^ (≈560 cm^−1^) and Ce^3+^O_7_V^●●^ (≈580 cm^−1^). The present results point to a non-negligible concentration of Ce^3+^ ions in NPs.

Moving to panel e, the Ce900 sample features only an overtone 2LO peak centered at 1168 cm^−1^, while spectra become progressively complex on reducing the crystal dimension. In particular, two sharp peaks, labelled with asterisks, appear at 825 and 930 cm^−1^; the overtone 2LO peak broaden and additional peaks appear at both sides. In the Ce200 sample, Raman peaks are centered at 1034, 1178, 1339 and 1506 cm^−1^. Raman peaks around 830–840 cm^−1^ [91,93,98] and 930 cm^−1^ [91] are attributed to peroxo $O_{2}^{2-}$ ions. Peaks in the 1000–1600 cm^−1^ range are attributed to $O_{2}^{\delta -}$(0 < δ < 1) adsorbed species [91,102]. In particular, the superoxide ions $O_{2}^{-}$ signal should drop around 1140 cm^−1^ [89,91], superimposed to the overtone 2LO. Raman results corroborate the ESR findings, pointing to a great oxygen reduction activity of CeO_2_ NPs produced by the adopted inverse micellae method.

Panel f reports the spectral range of 2500–4000 cm^−1^. Two intense peaks appear at ≈2850 and ≈2935 cm^−1^ in the spectrum of the Ce200 sample, attributable to the stretching of C-H bonds. The former reduces drastically its intensity for specimen Ce500, while the latter seems not to be affected by the higher annealing T. Finally, for the Ce900 sample, the intensity of both peaks is strongly reduced. We attribute tentatively these two peaks to residual n-buthanol [103] and CTAB molecules [104], which were not eliminated by washing cycles with EtOH and water. Finally, the highest energy part of the spectrum is dominated by the O-H vibrations. The broad multicomponent peak centered around 3400–3600 cm^−1^ in Ce200 and Ce500 samples is attributed to OH from physisorbed H_2_O molecules [105,106]. This broad feature is followed in the Ce200 case by two sharp peaks (labelled by asterisks) at 3650 and 3680 cm^−1^, which correspond to stretching of hydroxyl groups [106]. The same peaks reduce to a shoulder in the Ce500 sample, while a broad bump is barely detectable for Ce900.

Remembering that the synthesis is carried on in alkaline environments, chemisorbed hydroxyl groups partially cover the NP surface in the Ce200 sample. Firing at 500 °C removes these groups, so that only physisorbed water coming from moisture appears in the Ce500 sample, since measurements are made in air. In sample Ce900, the surface to bulk ratio is so small that even the contribution of OH vibrations in surface H_2_O is barely detectable in the Raman spectrum.

### 3.4. Atomistic Simulations

The largest atomistic model of the nanoparticle is made up of 1848 atoms of perfectly stoichiometric ceria (Ce_616_O_1232_), before adding dissociated water to its surface.

**Figure 9:**
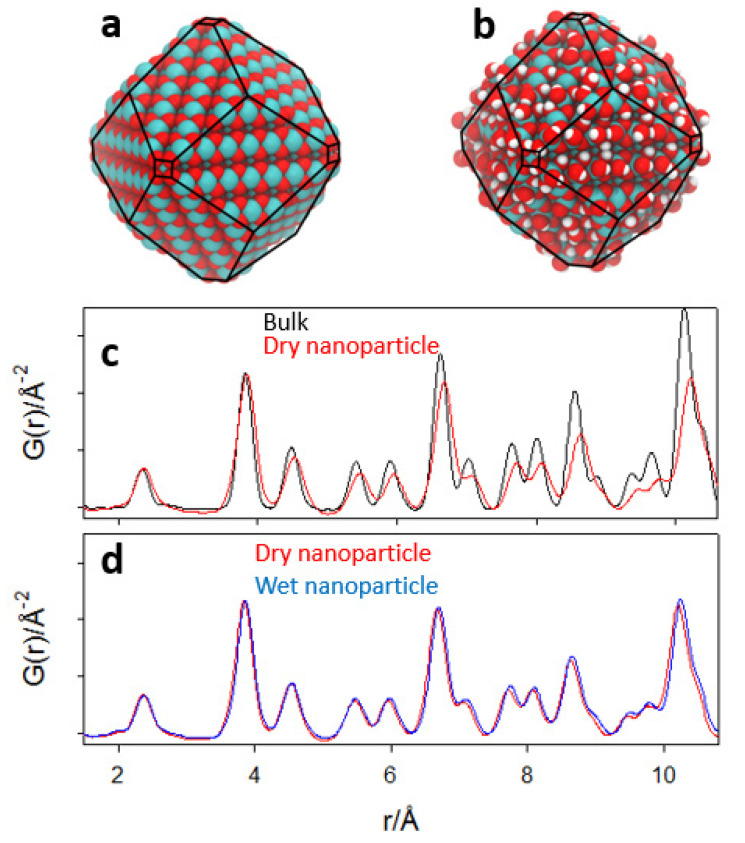
Force field computed dry (**a**) and wet (**b**) relaxed nanoparticles. (**c**,**d**) computed G(*r*) curves of bulk (black curve), dry (red curve) and wet (blue curve) nanoparticles.

Its largest linear dimension is approximately 40 Å, and its shape is a rhombic dodecahedron, exposing large {110} buckled surfaces and very small {100} facets on the corner, according to the Wulff construction (see Figure 9a). The corresponding wet nanoparticle is made up of 2232 atoms, i.e., with an additional 128 dissociated water molecules (shown in Figure S1b). This last model aims at miming the large concentration of hydroxyl groups revealed by Raman spectroscopy.

The prevalence of wet {110} surfaces is due to their low formation energy and is in agreement with experimental observations. In this case, the Wulff construction cannot produce elongated NPs, since it does not take into account temperature and kinetic effects, which can favor the growth of the NPs in preferential directions.

For the sake of comparison to the experimental data, we simulated the PDF of the atomistic NP models. First, the bulk CeO_2_ was relaxed according to the force field calculations; then, the NP was cut following the Wulff construction; G(*r*) curves were then computed before and after relaxation and after adsorption of water. The aim was to analyze separately: (1) the effect of the relaxation of the NP cut from the bulk; (2) the effect of water adsorption. As reported in [64], the force field has a false local minimum, involving a short Ce-O bond (≈1.9 Å) at under-coordinated Ce sites at the surface. Therefore, in this nanoparticle, we excluded the 218 Ce atoms in simulating the PDF.

In Figure 9c, the dry NPs are compared before (i.e., cut from the ideal fluorite structure) and after geometry relaxation. In Figure 9d, the calculated PDFs of the relaxed models of dry and wet NPs are compared. To ease comparisons, the G(*r*) curves are shown after slope subtraction. Our results show that the formation of the NP facets is accompanied by a substantial change in the interatomic distances. The PDF peaks are broadened due to a larger configurational disorder, and the peaks shift at larger *r*-values (Figure 9c). Water adsorption increases further disorder and expands interatomic distances, although not as much as the former relaxations (Figure 9d). The bond length expansion in NPs is in fairly good agreement with the evolution of sample Ce900, where the average size of the NP is large, and Ce200, where the size of the NP is similar to the atomistic models.

**Figure 10:**
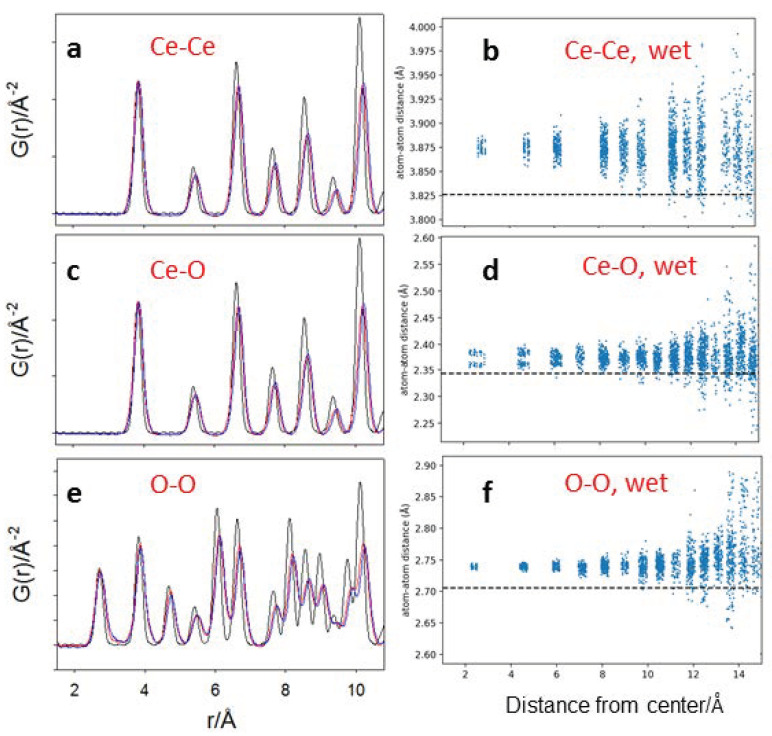
(**a**,**c**,**e**) Force field computed Ce–Ce, Ce–O and O–O partial PDFs, respectively. The colors of panels (**c**,**d**) are the same as Figure 9. The right-hand panels report the shortest Ce–Ce (**b**), Ce-O (**d**) and O–O (**f**) distances as a function of the distance of the couples of atoms from the center of the wet nanoparticle. Dashed black lines indicate the optimized Ce–Ce, Ce–O and O–O distances for relaxed bulk CeO_2_.

To further analyze the relaxation of the interatomic distance, Figure 10 reports the Ce–Ce, Ce–O and O–O partial PDFs, i.e., the PDF corresponding to single atom pair distances. In the left panels (Figure 10a,c,e), the partial PDFs are displayed, while in the right panels (Figure 10b,d,f), the pair distances are plotted as a function of the distance from the center of the relaxed model of the wet nanoparticle. Partial G(*r*)s evidence that the oxygen pattern is more disordered than the Ce in the NPs, in accordance with PDF analysis. In addition, the atomic relaxation of the NPs follows a complex pattern: the spread of interatomic distances increases with the distance from the center. The relaxation of the facets begins to be visible above 14–15 Å from the center, pointing to structural disorder located mainly at the surface.

It should be noted that the plots of Figure 10b,d,f do not evidence bond distance expansion at the surface, at least for the shortest interatomic vectors. Indeed, there are significant differences between the physics of the NPs unveiled by experimental probes and the (necessary simpler) modelling herein described. Besides the spherical envelope constrain of the Wulff construction and the limits of the force field evidenced above, the NP is constrained to be stoichiometric. Conversely, Raman spectroscopy clearly evidenced the presence of oxygen vacancies, especially in small NPs, and of partially reduced oxygen species, such us the superoxide ion $O_{2}^{-}$ confirmed also by ESR. Such an activity allows locating Ce^3+^/Ce^4+^ redox couples at the surface. We plan to overcome these limitations in a future article, where NPs will be computed using DFT, and explicitly oxygen vacancies will be introduced into the structure.

## 4. Discussion and Conclusions

In this paper, we presented a diffractometric, spectroscopic and computational study on ultrasmall CeO_2_ nanoparticles produced using an inverse micellae wet synthetic path followed by heating in air at 200 °C. To highlight the effect of nanostructuring in respect to other variables, aliquots of the same batch were fired at higher temperatures.

Findings of different techniques and data analysis approaches bring complementary pieces of information, allowing an exhaustive characterization. Starting from diffraction, traditional data analysis tools such as the Williamson Hall and the Rietveld refinements have identified some structural trends as a function of the crystal dimensions. In small NPs, the cell constant, the microstrain and the atomic mean square displacements enlarge in respect to bulk materials. Since the surface-to-bulk ratio is proportional to $D_{V}^{-1}$ (at least in the frame of monodisperse spherical particles), these results point to some surface relaxation and disordering.

To obtain further insight, we moved from the analysis of the Bragg peaks to total scattering approaches, namely DSE and PDF analysis. DSE focused mainly on the sample annealed at 200 °C and revealed that diffraction data are suitably fitted using a bi-layer prismatic core-shell model exposing {011} and {100} facets, where the shell is expanded (*a_shell_* = 5.424 Å) in respect to the core (*a_core_* = 5.386 Å), with a relative shell-to-core expansion of 0.7%. Moreover, Ce and O located in the outer expanded shell show larger thermal displacements compared to those in the core.

In addition, PDF analysis pointed to expansion of interatomic distances, especially at large *r* values. Although the real-space Rietveld model is constrained for simplicity to monodisperse spherical particles, good fits are obtained even at the shortest *r* distances, suggesting that the surface structure reconstruction preserves the fluorite architecture. Box-car refinements revealed growing trends of both the cell constant and the displacement parameters, which may be rationalized using the core-shell scenario of DSE analysis. In fact, very similar trends of the same parameters are observed in the box-car analysis of PDFs derived from diffraction patterns computed starting from the DSE model. It should be noted that differences in the absolute values (e.g., of the a parameter) are due to the different temperatures of the DSE (RT) and PDF (90 K) experiments.

Both DSE and PDF (box car) analysis point to the same core-shell model, evidencing different features. On one hand, DSE reveals the (ultrasmall) nanoparticle shape and core-shell nature; on the other hand, PDF describes the average values and distribution functions of different interatomic distances as a function of interatomic distances and of particle dimensions. Although both methods make use of the same experimental features (Bragg peaks and diffuse scattering), they evidence different outcomes and should be considered complementary to each other, due to the different conditions under which the two experiments are performed (high *Q*-max for PDF vs. high *Q*-resolution for DSE).

We also underline the added value of the total scattering analysis in respect to the ones that make use only of Bragg peaks to map fundamental structural features of NPs. Comparing the WH and Rietveld results on different samples suggests that the increased microstrain, cell volume and thermal parameters of the smallest NPs (Ce200) originate from structural relaxations at the surface. Conversely, DSE and PDF analysis supply a quantitative picture of the actual nanoparticle shape and the extent of relaxation at the surface.

Spectroscopic techniques bring some complementary insights on the nanoparticle and nanoparticle surface structures. Whereas the micro-Raman feature(s) in the 500–600 cm^−1^ point to a larger concentration of oxygen vacancies in respect to bigger particles, high frequency modes reveal the presence of hydroxyl and reduced oxygen species, such as $O_{2}^{-}$ at the surface. The last is confirmed also by ESR spectroscopy.

Theoretical computations deepen in the mechanism of structural relaxation. It is shown that both surface relaxation and wetting have a role in constructing the final structure of the nanoparticle, with the former effect being more effective in this purpose. Moreover, the analysis of the first Ce–O, Ce–Ce and O–O distances versus the distance of the atomic couples from the center of the nanoparticle confirms that disorder is boosted at the surface. However, the comparison between experiments and calculation is somewhat limited by the constraints derived by the (state-of-the-art) force field adopted, including the Ce_n_O_2n_ stoichiometry. Discrepancies among them suggest that oxygen vacancies (and Ce^3+^ species, neglected in calculations) should have a role in the structural expansion at the surface. We plan to overcome these limitations in a future paper, where NPs will be computed using DFT and explicitly oxygen vacancies will be introduced into the structure.

The previous discussion aimed to highlight the structural deviations of ultrathin NPs in respect to the bulk material features. In this respect, results on the larger particles (D_V_ ≈ 10 and ≈230 nm) were presented, essentially for the sake of comparison. However, cerium oxide particles are suitable for many different applications, and in each case, some “ideal” particle dimension fits the desired structural features. In this contest, the high *T* in situ PDF results supply some insights on the particles’ structural evolution during the firing process and help to determine the most suitable annealing path. The trends of displacement parameters and crystal size revealed that the high *T* treatments progressively reduce disorder while enlarging the particle dimensions. In particular, between 300 and 500 °C, where U(O) and especially U(Ce) display a decreasing trend, it is a clear fingerprint of progressive reduction of structural disorder. At higher temperatures, the displacement parameters invert their trend, and the crystal growth rate is boosted.

## Figures and Tables

**Table 1:** Outcomes of Rietveld refinements and WH analysis as a function of the annealing temperature.

Sample Name	Ce200	Ce400	Ce500	Ce700	Ce900
T_ann_/°C	200	400	500	700	900
Space group	$Fm\bar{3}m$	$Fm\bar{3}m$	$Fm\bar{3}m$	$Fm\bar{3}m$	$Fm\bar{3}m$
*a*/Å	5.4174(2)	5.40730(7)	5.4056(4)	5.40501(4)	5.40512(2)
U(Ce)/Å^2^	0.0047(2)	0.00234(8)	0.00193(7)	0.00154(5)	0.00155(6)
U(O)/Å^2^	0.0135(5)	0.0039(3)	0.0045(3)	0.0036(3)	0.0036(4)
Rp	0.0240	0.0332	0.0335	0.0443	0.0545
R(F^2^)	0.0098	0.0136	0.0133	0.0255	0.0304
D_V_/nm	3.1(3)	6.9(3)	9.7(3)	57(2)	227(8)
ε	0.007(2)	0.0028(4)	0.0017(2)	0.00030(3)	0.000020(8)

**Table 2:** Comparison among the models of CeO_2_ NPs tested by DSE against the XRPD pattern of the Ce200 specimen. All the sizes are given as mass-based average values.

Model	Spherical	Spherical Core-Shell	Prismatic	Prismatic Core-Shell
*a*/Å	5.4155	core 5.4050 shell 5.4360	5.4162	core 5.3850 shell 5.4240
<D_ab_> (nm)	3.31	3.34	3.66	3.60
σ/<D_ab_>	0.24	0.30	0.15	0.25
<L_a_> = <L_b_> (nm)	-	-	3.85	2.61
σ/<L>	-	-	0.21	0.32
<L_c_> (nm)	-	-	1.77	3.88
σ/<L>	-	-	0.19	0.38
U(O)_core_/Å^2^	0.005	0.005	0.017	0.005
U(O)_shell_/Å^2^	0.011	0.012	0.005	0.011
U(Ce)_core_/Å^2^	0.005	0.005	0.005	0.005
U(Ce)_shell_/Å^2^	0.024	0.022	0.012	0.007
wR%	6.47	5.92	5.23	4.32
GoF	15.54	14.99	12.35	10.94

**Table 3:** Parameters adopted to fit the ESR spectra. Gw_pp_ and Lw_pp_ are FWHM of the Gaussian and Lorentian contributions to peaks broadening; D and E are zero field splitting parameters; and C_Gd_ and C_O_ are the multiplicative (scale) coefficients introduced in EasySpin for Gadolinium and Oxygen species, respectively.

Sample		Ce200	Ce500	Ce900
Feature A Gd^3+^	$g_{⊥}$	1.975	1.975	1.983
	$g_{∥}$	1.950	1.950	1.960
	$Gw_{PP}$/mT	-	-	-
	$Lw_{PP}$/mT	0.5	0.5	0.5
	D/MHz	126	126	126
	E/MHz	37.8	37.8	37.8
	C_Gd_	1.5	1.5	1.5
Feature B $O_{2}^{-}$	$g_{⊥}$	2.212	2.218	-
	$g_{∥}$	2.244	2.186	-
	$Gw_{PP}$/mT	35.9	27.4	-
	$Lw_{PP}$/mT	62.9	87.0	-
	C_O_	12500	4500	-

## Data Availability

The DebUsSy program suite is freely available at https://debyeusersystem.github.io accessed on 24 August 2022.

## Supplementary Materials

The following supporting information can be downloaded at: https://www.mdpi.com/article/10.3390/nano12193385/s1, Text S1: DSE methods; Figure S1: Prismatic nanocrystals faceting; Figure S2: DSE analysis of sample Ce500; Table S1: ESR spectra parameters.

## References

[1] Rietveld H.M. (1969). A Profile Refinement Method for Nuclear and Magnetic Structures. J. Appl. Crystallogr..

[2] Billinge S.J.L., Kanatzidis M.G. (2004). Beyond Crystallography: The Study of Disorder, Nanocrystallinity and Crystallographically Challenged Materials with Pair Distribution Functions. Chem. Commun..

[3] Billinge S.J.L., Levin I. (2007). The Problem with Determining Atomic Structure at the Nanoscale. Science.

[4] Egami T., Billinge S.J.L. (2012). Underneath the Bragg Peaks: Structural Analysis of Complex Materials.

[5] Chupas P.J., Chapman K.W., Chen H., Grey C.P. (2009). Application of High-Energy X-rays and Pair-Distribution-Function Analysis to Nano-Scale Structural Studies in Catalysis. Catal. Today.

[6] Toby B.H., Egami T. (1992). Accuracy of Pair Distribution Function Analysis Applied to Crystalline and Non-Crystalline Materials. Acta Cryst. A.

[7] Zhu H., Huang Y., Ren J., Zhang B., Ke Y., Jen A.K.-Y., Zhang Q., Wang X.-L., Liu Q. (2021). Bridging Structural Inhomogeneity to Functionality: Pair Distribution Function Methods for Functional Materials Development. Adv. Sci..

[8] Bertolotti F., Protesescu L., Kovalenko M.V., Yakunin S., Cervellino A., Billinge S.J.L., Terban M.W., Pedersen J.S., Masciocchi N., Guagliardi A. (2017). Coherent Nanotwins and Dynamic Disorder in Cesium Lead Halide Perovskite Nanocrystals. ACS Nano.

[9] Bertolotti F., Moscheni D., Guagliardi A., Masciocchi N. (2018). When Crystals Go Nano—The Role of Advanced X-ray Total Scattering Methods in Nanotechnology. Eur. J. Inorg. Chem..

[10] Moscheni D., Bertolotti F., Piveteau L., Protesescu L., Dirin D.N., Kovalenko M.V., Cervellino A., Pedersen J.S., Masciocchi N., Guagliardi A. (2018). Size-Dependent Fault-Driven Relaxation and Faceting in Zincblende CdSe Colloidal Quantum Dots. ACS Nano.

[11] Bertolotti F., Dirin D.N., Ibáñez M., Krumeich F., Cervellino A., Frison R., Voznyy O., Sargent E.H., Kovalenko M.V., Guagliardi A. (2016). Crystal Symmetry Breaking and Vacancies in Colloidal Lead Chalcogenide Quantum Dots. Nat. Mater..

[12] Dengo N., Masciocchi N., Cervellino A., Guagliardi A., Bertolotti F. (2022). Effects of Structural and Microstructural Features on the Total Scattering Pattern of Nanocrystalline Materials. Nanomaterials.

[13] Trovarelli A. (1996). Catalytic Properties of Ceria and CeO_2_-Containing Materials. Catal. Rev..

[14] Trovarelli A. (1999). Structural and Oxygen Storage/Release Properties of CeO_2_-Based Solid Solutions. Comments Inorg. Chem..

[15] Su Y.-Q., Filot I.A.W., Liu J.-X., Tranca I., Hensen E.J.M. (2016). Charge Transport over the Defective CeO_2_(111) Surface. Chem. Mater..

[16] Tuller H.L., Nowick A.S. (1979). Defect Structure and Electrical Properties of Nonstoichiometric CeO_2_ Single Crystals. J. Electrochem. Soc..

[17] Mogensen M., Sammes N.M., Tompsett G.A. (2000). Physical, Chemical and Electrochemical Properties of Pure and Doped Ceria. Solid State Ion..

[18] Coduri M., Checchia S., Longhi M., Ceresoli D., Scavini M. (2018). Rare Earth Doped Ceria: The Complex Connection Between Structure and Properties. Front. Chem..

[19] Han X., Lee J., Yoo H.-I. (2009). Oxygen-Vacancy-Induced Ferromagnetism in CeO_2_ from First Principles. Phys. Rev. B.

[20] Marrocchelli D., Bishop S.R., Tuller H.L., Watson G.W., Yildiz B. (2012). Charge Localization Increases Chemical Expansion in Cerium-Based Oxides. Phys. Chem. Chem. Phys..

[21] Oliva C., Scavini M., Ballabio O., Sin A., Zaopo A., Dubitsky Y. (2004). Percolative Small-Polarons Conduction Regime in Ce_1−x_Gd_x_O_2−x/2_, Probed by the EPR Spectral Intensity of Gd^3+^. J. Solid State Chem..

[22] Lavik E.B., Kosacki I., Tuller H.L., Chiang Y.-M., Ying J.Y. (1997). Nonstoichiometry and Electrical Conductivity of Nanocrystalline CeO_2−X_. J. Electroceramics.

[23] Bruix A., Neyman K.M. (2016). Modeling Ceria-Based Nanomaterials for Catalysis and Related Applications. Catal. Lett..

[24] Kung M.C., Ye J., Kung H.H. (2019). 110th Anniversary: A Perspective on Catalytic Oxidative Processes for Sustainable Water Remediation. Ind. Eng. Chem. Res..

[25] Trovarelli A., Llorca J. (2017). Ceria Catalysts at Nanoscale: How Do Crystal Shapes Shape Catalysis?. ACS Catal..

[26] Melchionna M., Fornasiero P. (2014). The Role of Ceria-Based Nanostructured Materials in Energy Applications. Mater. Today.

[27] Teh L.P., Setiabudi H.D., Timmiati S.N., Aziz M.A.A., Annuar N.H.R., Ruslan N.N. (2021). Recent Progress in Ceria-Based Catalysts for the Dry Reforming of Methane: A Review. Chem. Eng. Sci..

[28] Ricote S., Jacobs G., Milling M., Ji Y., Patterson P.M., Davis B.H. (2006). Low Temperature Water—Gas Shift: Characterization and Testing of Binary Mixed Oxides of Ceria and Zirconia Promoted with Pt. Appl. Catal. A Gen..

[29] Razmgar K., Altarawneh M., Oluwoye I., Senanayake G. (2021). Ceria-Based Catalysts for Selective Hydrogenation Reactions: A Critical Review. Catal. Surv. Asia.

[30] Xie S., Wang Z., Cheng F., Zhang P., Mai W., Tong Y. (2017). Ceria and Ceria-Based Nanostructured Materials for Photoenergy Applications. Nano Energy.

[31] Li H., Xia P., Pan S., Qi Z., Fu C., Yu Z., Kong W., Chang Y., Wang K., Wu D. (2020). The Advances of Ceria Nanoparticles for Biomedical Applications in Orthopaedics. Int. J. Nanomed..

[32] Trovarelli A. (2002). Catalysis by Ceria and Related Materials. Catalytic Science Series.

[33] Bevan D.J.M., Kordis J. (1964). Mixed Oxides of the Type MO_2_ (Fluorite)—M_2_O_3_—I Oxygen Dissociation Pressures and Phase Relationships in the System CeO_2_ Ce_2_O_3_ at High Temperatures. J. Inorg. Nucl. Chem..

[34] Kümmerle E.A., Heger G. (1999). The Structures of C–Ce_2_O_3+δ_, Ce_7_O_12_, and Ce_11_O_20_. J. Solid State Chem..

[35] Mamontov E., Egami T., Brezny R., Koranne M., Tyagi S. (2000). Lattice Defects and Oxygen Storage Capacity of Nanocrystalline Ceria and Ceria-Zirconia. J. Phys. Chem. B.

[36] Luo S., Li M., Fung V., Sumpter B.G., Liu J., Wu Z., Page K. (2021). New Insights into the Bulk and Surface Defect Structures of Ceria Nanocrystals from Neutron Scattering Study. Chem. Mater..

[37] Cresi J.S.P., Spadaro M.C., D’Addato S., Valeri S., Amidani L., Boscherini F., Bertoni G., Deiana D., Luches P. (2017). Contraction, Cation Oxidation State and Size Effects in Cerium Oxide Nanoparticles. Nanotechnology.

[38] Baranchikov A.E., Polezhaeva O.S., Ivanov V.K., Tretyakov Y.D. (2010). Lattice Expansion and Oxygen Non-Stoichiometry of Nanocrystalline Ceria. CrystEngComm.

[39] Kimmel G., Sahartov A., Sadia Y., Porat Z., Zabicky J., Dvir E. (2021). Non-Monotonic Lattice Parameters Variation with Crystal Size in Nanocrystalline CeO_2_. J. Mater. Res. Technol..

[40] Deshpande S., Patil S., Kuchibhatla S.V., Seal S. (2005). Size Dependency Variation in Lattice Parameter and Valency States in Nanocrystalline Cerium Oxide. Appl. Phys. Lett..

[41] Shannon R.D. (1976). Revised Effective Ionic Radii and Systematic Studies of Interatomic Distances in Halides and Chalcogenides. Acta Cryst. A.

[42] Coduri M., Brunelli M., Scavini M., Allieta M., Masala P., Capogna L., Fischer H.E., Ferrero C. (2012). Rare Earth Doped Ceria: A Combined X-ray and Neutron Pair Distribution Function Study. Z. Krist..

[43] Coduri M., Scavini M., Brunelli M., Masala P. (2013). In Situ Pair Distribution Function Study on Lanthanum Doped Ceria. Phys. Chem. Chem. Phys..

[44] Hayashi H., Sagawa R., Inaba H., Kawamura K. (2000). Molecular Dynamics Calculations on Ceria-Based Solid Electrolytes with Different Radius Dopants. Solid State Ion..

[45] Sathyamurthy S., Leonard K.J., Dabestani R.T., Paranthaman M.P. (2005). Reverse Micellar Synthesis of Cerium Oxide Nanoparticles. Nanotechnology.

[46] Fitch A.N. (2004). The High Resolution Powder Diffraction Beam Line at ESRF. J. Res. Natl. Inst. Stand. Technol..

[47] Dejoie C., Coduri M., Petitdemange S., Giacobbe C., Covacci E., Grimaldi O., Autran P.-O., Mogodi M.W., Šišak Jung D., Fitch A.N. (2018). Combining a Nine-Crystal Multi-Analyser Stage with a Two-Dimensional Detector for High-Resolution Powder X-ray Diffraction. J. Appl. Cryst..

[48] Williamson G.K., Hall W.H. (1953). X-ray Line Broadening from Filed Aluminium and Wolfram. Acta Metall..

[49] Larson A.C., Von Dreele R.B. (2004). General Structure Analysis System (GSAS).

[50] Toby B.H. (2001). EXPGUI, a Graphical User Interface for GSAS. J. Appl. Cryst..

[51] Vaughan G.B., Baker R., Barret R., Bonnefoy J., Buslaps T., Checchia S., Duran D., Fihman F., Got P., Kieffer J. (2020). ID15A at the ESRF–a Beamline for High Speed Operando X-ray Diffraction, Diffraction Tomography and Total Scattering. J. Synchrotron Radiat..

[52] Kieffer J., Wright J.P. (2013). PyFAI: A Python Library for High Performance Azimuthal Integration on GPU. Powder Diffr..

[53] Juhás P., Davis T., Farrow C.L., Billinge S.J.L. (2013). PDFgetX3: A Rapid and Highly Automatable Program for Processing Powder Diffraction Data into Total Scattering Pair Distribution Functions. J. Appl. Cryst..

[54] Farrow C.L., Juhas P., Liu J.W., Bryndin D., Božin E.S., Bloch J., Proffen T., Billinge S.J.L. (2007). PDFfit2 and PDFgui: Computer Programs for Studying Nanostructure in Crystals. J. Phys. Condens. Matter.

[55] Willmott P.R., Meister D., Leake S.J., Lange M., Bergamaschi A., Böge M., Calvi M., Cancellieri C., Casati N., Cervellino A. (2013). The Materials Science Beamline Upgrade at the Swiss Light Source. J. Synchrotron Radiat..

[56] Bergamaschi A., Cervellino A., Dinapoli R., Gozzo F., Henrich B., Johnson I., Kraft P., Mozzanica A., Schmitt B., Shi X. (2010). The MYTHEN Detector for X-ray Powder Diffraction Experiments at the Swiss Light Source. J. Synchrotron Radiat..

[57] Bowden M., Ryan M. (2010). Absorption Correction for Cylindrical and Annular Specimens and Their Containers or Supports. J. Appl. Cryst..

[58] Stoll S., Schweiger A. (2006). EasySpin, a Comprehensive Software Package for Spectral Simulation and Analysis in EPR. J. Magn. Reson..

[59] (2012). MATLAB and Statistics Toolbox Release 2012.

[60] Giannozzi P., Baroni S., Bonini N., Calandra M., Car R., Cavazzoni C., Ceresoli D., Chiarotti G.L., Cococcioni M., Dabo I. (2009). QUANTUM ESPRESSO: A Modular and Open-Source Software Project for Quantum Simulations of Materials. J. Phys. Condens. Matter.

[61] Giannozzi P., Andreussi O., Brumme T., Bunau O., Nardelli M.B., Calandra M., Car R., Cavazzoni C., Ceresoli D., Cococcioni M. (2017). Advanced Capabilities for Materials Modelling with Quantum ESPRESSO. J. Phys. Condens. Matter.

[62] Nielsen M.B., Ceresoli D., Jørgensen J.-E., Prescher C., Prakapenka V.B., Bremholm M. (2017). Experimental Evidence for Pressure-Induced First Order Transition in Cerium Nitride from B1 to B10 Structure Type. J. Appl. Phys..

[63] Mathew K., Singh A.K., Gabriel J.J., Choudhary K., Sinnott S.B., Davydov A.V., Tavazza F., Hennig R.G. (2016). MPInterfaces: A Materials Project Based Python Tool for High-Throughput Computational Screening of Interfacial Systems. Comput. Mater. Sci..

[64] Broqvist P., Kullgren J., Wolf M.J., van Duin A.C.T., Hermansson K. (2015). ReaxFF Force-Field for Ceria Bulk, Surfaces, and Nanoparticles. J. Phys. Chem. C.

[65] van Duin A.C.T., Dasgupta S., Lorant F., Goddard W.A. (2001). ReaxFF:  A Reactive Force Field for Hydrocarbons. J. Phys. Chem. A.

[66] Aryanpour M., van Duin A.C.T., Kubicki J.D. (2010). Development of a Reactive Force Field for Iron−Oxyhydroxide Systems. J. Phys. Chem. A.

[67] Thompson A.P., Aktulga H.M., Berger R., Bolintineanu D.S., Brown W.M., Crozier P.S., in ’t Veld P.J., Kohlmeyer A., Moore S.G., Nguyen T.D. (2022). LAMMPS—A Flexible Simulation Tool for Particle-Based Materials Modeling at the Atomic, Meso, and Continuum Scales. Comput. Phys. Commun..

[68] Scavini M., Coduri M., Allieta M., Masala P., Cappelli S., Oliva C., Brunelli M., Orsini F., Ferrero C. (2015). Percolating Hierarchical Defect Structures Drive Phase Transformation in Ce_1−*x*_Gd*_x_*O_2−*x*/2_: A Total Scattering Study. Int. Union Crystallogr..

[69] Scavini M., Coduri M., Allieta M., Brunelli M., Ferrero C. (2012). Probing Complex Disorder in Ce_1−*x*_Gd*_x_*O_2−*x*/2_ Using the Pair Distribution Function Analysis. Chem. Mater..

[70] Zhao Y., Xing W., Meng F., Sha H., Yu Y., Ma X., Yu R. (2021). Metastable Ce-Terminated (111) Surface of Ceria. Appl. Surf. Sci..

[71] Argyriou D.N. (1994). Measurement of the Static Disorder Contribution to the Temperature Factor in Cubic Stabilized ZrO_2_. J. Appl. Cryst..

[72] Coduri M., Scavini M., Allieta M., Brunelli M., Ferrero C. (2012). Local Disorder in Yttrium Doped Ceria (Ce_1−x_Y_x_O_2−x/2_) Probed by Joint X-ray and Neutron Powder Diffraction. J. Phys. Conf. Ser..

[73] Palosz B., Grzanka E., Gierlotka S., Stelmakh S., Pielaszek R., Bismayer U., Neuefeind J., Weber H.-P., Palosz W. (2000). Diffraction Studies of Nanocrystals: Theory and Experiment. Acta Phys. Pol. A.

[74] Zhou X.-D., Huebner W. (2001). Size-Induced Lattice Relaxation in CeO_2_ Nanoparticles. Appl. Phys. Lett..

[75] Zhang F., Chan S.-W., Spanier J.E., Apak E., Jin Q., Robinson R.D., Herman I.P. (2002). Cerium Oxide Nanoparticles: Size-Selective Formation and Structure Analysis. Appl. Phys. Lett..

[76] Mai H.-X., Sun L.-D., Zhang Y.-W., Si R., Feng W., Zhang H.-P., Liu H.-C., Yan C.-H. (2005). Shape-Selective Synthesis and Oxygen Storage Behavior of Ceria Nanopolyhedra, Nanorods, and Nanocubes. J. Phys. Chem. B.

[77] Cervellino A., Frison R., Bertolotti F., Guagliardi A. (2015). DEBUSSY 2.0: The New Release of a Debye User System for Nanocrystalline and/or Disordered Materials. J. Appl. Crystallogr..

[78] Coduri M., Scavini M., Allieta M., Brunelli M., Ferrero C. (2013). Defect Structure of Y-Doped Ceria on Different Length Scales. Chem. Mater..

[79] Howell R.C., Proffen T., Conradson S.D. (2006). Pair Distribution Function and Structure Factor of Spherical Particles. Phys. Rev. B.

[80] Qiu X., Božin E.S., Juhas P., Proffen T., Billinge S.J.L. (2004). Reciprocal-Space Instrumental Effects on the Real-Space Neutron Atomic Pair Distribution Function. J. Appl. Cryst..

[81] Abi-aad E., Bechara R., Grimblot J., Aboukais A. (1993). Preparation and Characterization of Ceria under an Oxidizing Atmosphere. Thermal Analysis, XPS, and EPR Study. Chem. Mater..

[82] Li Z., Werner K., Qian K., You R., Płucienik A., Jia A., Wu L., Zhang L., Pan H., Kuhlenbeck H. (2019). Oxidation of Reduced Ceria by Incorporation of Hydrogen. Angew. Chem. Int. Ed..

[83] Oliva C., Forni L., Ezerets A.M., Mukovozov I.E., Vishniakov A.V. (1998). EPR Characterisation of (CeO_2_)_1−y_(La_2_CuO_4_)_y_ Oxide Mixtures and Their Catalytic Activity for NO Reduction by CO. J. Chem. Soc. Faraday Trans..

[84] Oliva C., Termignone G., Vatti F.P., Forni L., Vishniakov A.V. (1996). Electron Paramagnetic Resonance Spectra of CeO_2_ Catalyst for CO Oxidation. J. Mater. Sci..

[85] Figaj M., Becker K.D. (2001). An Electron Paramagnetic Resonance Study of Impurities in Ceria, CeO_2_. Solid State Ion..

[86] Wertz J.E., Bolton J.R. (1986). Electron Spin Resonance, Elementary Theory and Practical Applications.

[87] de Biasi R.S., Grillo M.L.N. (2005). Electron Spin Resonance of Diluted Solid Solutions of Gd_2_O_3_ in CeO_2_. J. Solid State Chem..

[88] Xu J., Harmer J., Li G., Chapman T., Collier P., Longworth S., Tsang S.C. (2010). Size Dependent Oxygen Buffering Capacity of Ceria Nanocrystals. Chem. Commun..

[89] Huang M., Fabris S. (2007). Role of Surface Peroxo and Superoxo Species in the Low-Temperature Oxygen Buffering of Ceria: Density Functional Theory Calculations. Phys. Rev. B.

[90] Pushkarev V.V., Kovalchuk V.I., d’Itri J.L. (2004). Probing Defect Sites on the CeO_2_ Surface with Dioxygen. J. Phys. Chem. B.

[91] Guzman J., Carrettin S., Corma A. (2005). Spectroscopic Evidence for the Supply of Reactive Oxygen during CO Oxidation Catalyzed by Gold Supported on Nanocrystalline CeO_2_. J. Am. Chem. Soc..

[92] Banerji A., Grover V., Sathe V., Deb S.K., Tyagi A.K. (2009). CeO_2_–Gd_2_O_3_ System: Unraveling of Microscopic Features by Raman Spectroscopy. Solid State Commun..

[93] Schilling C., Hofmann A., Hess C., Ganduglia-Pirovano M.V. (2017). Raman Spectra of Polycrystalline CeO_2_: A Density Functional Theory Study. J. Phys. Chem. C.

[94] Filtschew A., Hofmann K., Hess C. (2016). Ceria and Its Defect Structure: New Insights from a Combined Spectroscopic Approach. J. Phys. Chem. C.

[95] Dohčević-Mitrović Z.D., Šćepanović M.J., Grujić-Brojčin M.U., Popović Z.V., Bošković S.B., Matović B.M., Zinkevich M.V., Aldinger F. (2006). The Size and Strain Effects on the Raman Spectra of Ce_1−x_Nd_x_O_2−δ_ (0≤ x ≤0.25) Nanopowders. Solid State Commun..

[96] Nakajima A., Yoshihara A., Ishigame M. (1994). Defect-Induced Raman Spectra in Doped CeO_2_. Phys. Rev. B.

[97] McBride J.R., Hass K.C., Poindexter B.D., Weber W.H. (1994). Raman and X-ray Studies of Ce_1−x_RE_x_O_2−y_, Where RE=La, Pr, Nd, Eu, Gd, and Tb. J. Appl. Phys..

[98] Loridant S. (2021). Raman Spectroscopy as a Powerful Tool to Characterize Ceria-Based Catalysts. Catal. Today.

[99] Mochizuki S. (1982). Infrared Optical Properties of Cerium Dioxide. Phys. Status Solidi B.

[100] Coduri M., Scavini M., Pani M., Carnasciali M.M., Klein H., Artini C. (2017). From Nano to Microcrystals: Effects of Different Synthetic Pathways on the Defect Architecture in Heavily Gd-Doped Ceria. Phys. Chem. Chem. Phys..

[101] Artini C., Carnasciali M.M., Plaisier J.R., Costa G.A., Pani M. (2017). A Novel Method for the Evaluation of the Rare Earth (RE) Coordination Number in RE-Doped Ceria through Raman Spectroscopy. Solid State Ion..

[102] Long R.Q., Huang Y.P., Wan H.L. (1997). Surface Oxygen Species Over Cerium Oxide and Their Reactivities with Methane and Ethane by Means of in Situ Confocal Microprobe Raman Spectroscopy. J. Raman Spectrosc..

[103] Hédoux A., Guinet Y., Paccou L., Derollez P., Danède F. (2013). Vibrational and Structural Properties of Amorphous N-Butanol: A Complementary Raman Spectroscopy and X-ray Diffraction Study. J. Chem. Phys..

[104] Tyrode E., Rutland M.W., Bain C.D. (2008). Adsorption of CTAB on Hydrophilic Silica Studied by Linear and Nonlinear Optical Spectroscopy. J. Am. Chem. Soc..

[105] Uriarte L.M., Dubessy J., Boulet P., Baonza V.G., Bihannic I., Robert P. (2015). Reference Raman Spectra of Synthesized CaCl2 · NH2O Solids (n = 0, 2, 4, 6). J. Raman Spectrosc..

[106] Wang A., Freeman J.J., Jolliff B.L. (2015). Understanding the Raman Spectral Features of Phyllosilicates. J. Raman Spectrosc..

