# Supplementary material for: Structure and Surface Relaxation of CeO2 Nanoparticles Unveiled by Combining Real and Reciprocal Space Total Scattering Analysis
Source: Nanomaterials (Basel). 2022 Sep 27;12(19):3385. doi: 10.3390/nano12193385 (PMC9565251; doi:10.3390/nano12193385)
Supplement: Supplementary file 1 [file nanomaterials-12-03385-s001.zip › nanomaterials-1908095-supplementary.pdf]

Supporting Information

Structure and Surface Relaxation of CeO2 Nanoparticles Unveiled by Combining Real and Reciprocal Space Total Scattering Analysis

Marco Scavini*, Federica Bertolotti, Jonadri Mlloja, Filippo Umbri, Anna Bosc, Serena Cappelli, Stefano Checchia, Cesare Oliva, Patrizia Fumagalli, Davide Ceresoli, Mariangela Longhi, Antonella Guagliardi, and Mauro Coduri*

Text S1. The DSE modeling approach.

The DSE provides the average differential cross-section (or the powder diffraction pattern) of a randomly oriented powder from the distribution of interatomic distances between atomic pairs, without any assumption of periodicity and order [72]:

$$I(Q) = \sum_{j=1}^{N} f_j(Q)^2 o_j^2 + 2\sum_{j>i}^{N} f_j(Q) f_i(Q) T_j(Q) T_i(Q) o_j o_i \frac{\sin(Qd_{ij})}{(Qd_{ij})}$$

where Q = 4πsinθ/λ is the magnitude of the scattering vector, λ is the radiation wavelength, f_i is the atomic form factor of element i, d_{ij} is the interatomic distance between atoms i and j, N is the total number of atoms and T and o are the thermal atomic displacement parameter and the site occupancy factor associated to each atomic species, respectively. The first summation in the above equation includes the contributions of zero distances between one atom and itself and the second term (the interference term) the non-zero interatomic distances $d_{ij} = |r_i - r_j|$.

Spherical model. To compute the DSE according to the Debussy [72] strategy, first we built a monovariate population of atomistic models of CeO_2 nanocrystals (NCs) using the fluorite-type cubic structure. The DSE model computed from this population was then optimized against the experimental data, in terms of lognormal average size and size dispersion, lattice parameter, and isotropic atomic displacement parameters.

Spherical core-shell model. We developed a spherical core-shell atomistic model considering two configurations having different core/shell thickness ratios, with fixed shell thickness and refined core diameter. Configuration 1: shell corresponding to a single layer of lattice nodes (single fluorite-type unit cell, fixed thickness = 0.42 nm) surrounding the spherical core; Configuration 2: shell corresponding to

two layers of lattice nodes (two fluorite-type unit cells, fixed thickness = 0.84 nm) surrounding the spherical core.
A grid search was performed in order to explore the best a_{core} and a_{shell} parameters for the two configurations, in the range 5.390- 5.411 Å for the former and 5.420 – 5.445 Å for the latter, with a sampling step of 0.002 Å.

Prismatic models. We developed prismatic atomistic models according to bivariate populations of NCs breaking the cubic morphological symmetry. Three different structure-morphology configurations were considered. For each of them, the building block (unit cell) was chosen to account for different NCs growth directions and NCs faceting (see Figure S1 for the structure-morphology relationship of each configuration vs the reference cubic unit cell). Prismatic Configuration 1 (Figure S1a): cubic fluorite-type unit cell, NCs grown along the cubic [100] and [001] axes; exposed facets $\{100\}_k$.
Prismatic Configuration 2 (Figure S1b): trigonal lattice with unit cell vectors $\mathbf{a}_h = (-\mathbf{a}_k + \mathbf{b}_k)/2$; $\mathbf{b}_h = (-\mathbf{b}_k + \mathbf{c}_k)/2$; $\mathbf{c}_h = \mathbf{a}_k + \mathbf{b}_k + \mathbf{c}_k$; $a_h = b_h = \sqrt{2}/2 \cdot a_k$ and $c_h = \sqrt{3} \cdot a_k$), NCs grown along the cubic [111] and [-110]
Prismatic Configuration 3 (Figure S1c): tetragonal I-centred lattice with unit cell vectors $\mathbf{a}_t = \mathbf{a}_k$, $\mathbf{b}_t = (\mathbf{b}_k + \mathbf{c}_k)/2$ and $\mathbf{c}_t = (-\mathbf{b}_k + \mathbf{c}_k)/2$ and $b_t = c_t = \sqrt{2}/2 \cdot a_k$.

Prismatic core-shell model. For the prismatic model best matching the experimental data (Configuration 3) we develop a core-shell atomistic model considering two different core/shell thickness ratios, as detailed for the *Spherical core-shell model* (single shell /double shell layers of lattice nodes).
A grid search was performed in order to explore the best a_{core} and a_{shell} parameters for the two configurations, in the range 5.375 - 5.411 Å for the former and 5.415 – 5.430 Å for the latter, with a sampling step of 0.005 Å (next reduced to 0.002 Å around the minimum of the grid).

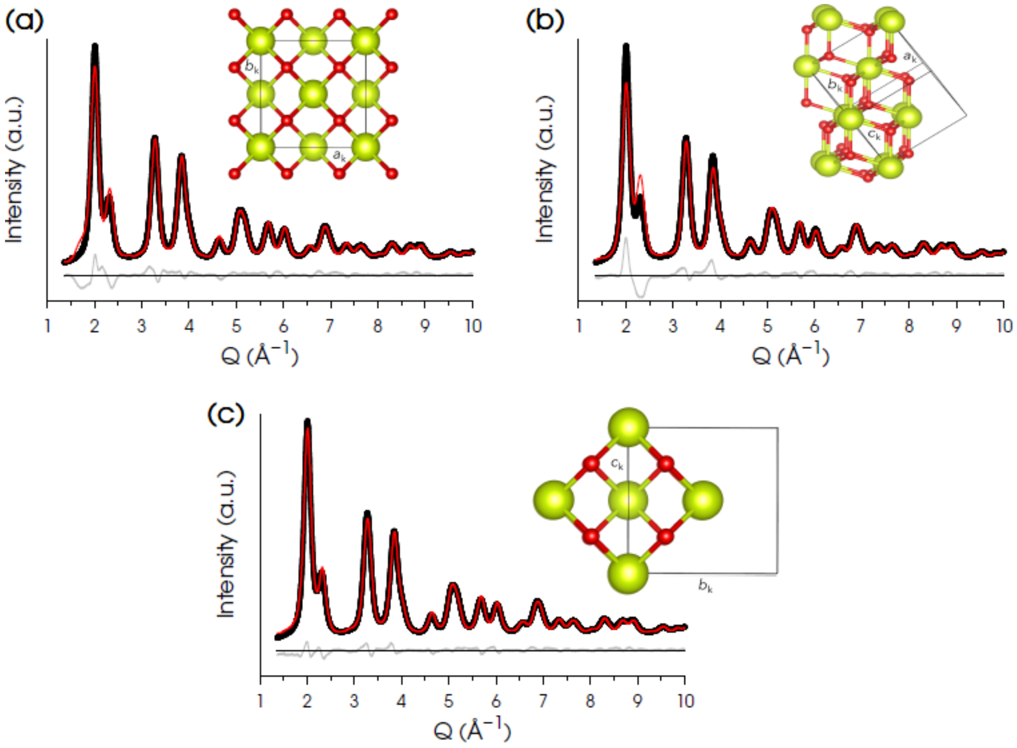

Figure S1. Prismatic nanocrystals faceting. Synchrotron XRPD data (black dots) of Ce200 and DSE best fits obtained using three different faceting for the prismatic nanocrystals displayed in the inset: **a)** cubic fluorite unit cell, nanocrystals grown along the [100] and [001] axes; exposed facets {100}; **b)** trigonal lattice with unit cell vectors $\mathbf{a}_h = (-\mathbf{a}_k + \mathbf{b}_k)/2$; $\mathbf{b}_h = (-\mathbf{b}_k + \mathbf{c}_k)/2$; $\mathbf{c}_h = \mathbf{a}_k + \mathbf{b}_k + \mathbf{c}_k$; $a_h = b_h = \sqrt{2}/2 \cdot a_k$ and $c_h = \sqrt{3} \cdot a_k$, nanocrystals grown along the [111] and [-110]; **c)** tetragonal I-centered lattice with unit cell vectors $\mathbf{a}_t = \mathbf{a}_k$, $\mathbf{b}_t = (\mathbf{b}_k + \mathbf{c}_k)/2$ and $\mathbf{c}_t = (-\mathbf{b}_k + \mathbf{c}_k)/2$ and $b_t = c_t = \sqrt{2}/2 \cdot a_k$. The geometrical relationship with the original cubic unit cell (in black) is also shown in the insets.

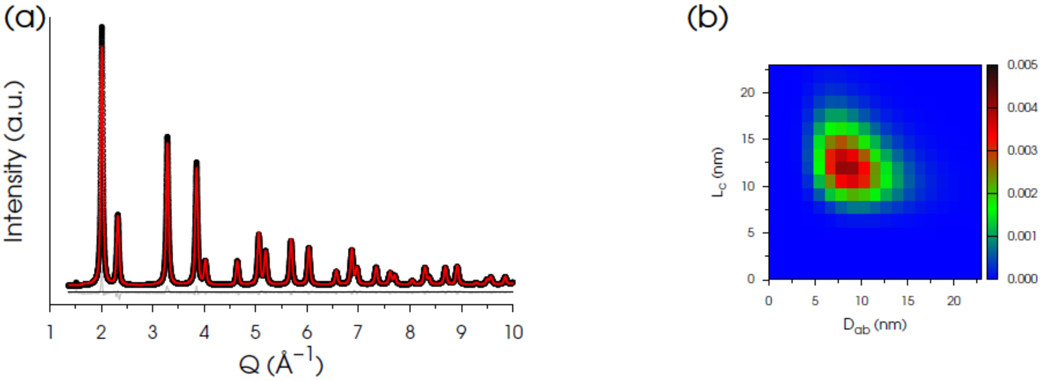

Figure S2. DSE analysis of Ce500. **a)** Synchrotron XRPD data (black dots) of Ce500 and DSE best fit obtained using {011} and {100} faceted CeO_2 nanocrystals with a fluorite-type structure and a uniform unit cell parameter (a = 5.4112 Å, GoF = 12.09). **b)** 2D map of the refined bivariate lognormal distribution in the D_{ab} (diameter of equivalent volume to the prism basal plane) and L_c coordinates.

Table S1. Parameters adopted to fit the ESR spectra. Gw_{pp} and Lw_{pp} are FWHM of the Gaussian and Lorentian contributions to peaks broadening; D and E are zero field splitting parameters and C_{Cr} and C_O are the multiplicative (scale) coefficients introduced in EasySpin for Chromium and Oxygen species, respectively.

Sample		Ce200	Ce500	Ce900
Feature A Cr^{3+}	$g_\perp$	1.975	1.975	1.983
	$g_\parallel$	1.950	1.950	1.960
	Gw_{PP}/mT	-	-	-
	Lw_{PP}/mT	0.5	0.5	0.5
	D/MHz	126	126	126
	E/MHz	37.8	37.8	37.8
	C_{Gd}	2.75	2.75	2.75
Feature B O_2^-	$g_\perp$	2.212	2.218	-
	$g_\parallel$	2.244	2.186	-
	Gw_{PP}/mT	35.9	27.4	-
	Lw_{PP}/mT	62.9	87.0	-
	C_O	12500	4500	-
